# Effects of a Four‐Strain Probiotic on Gut Microbiota, Inflammation, and Symptoms in Parkinson's Disease: A Randomized Clinical Trial

**DOI:** 10.1002/mds.70047

**Published:** 2025-10-23

**Authors:** Valentina Leta, Pavlos Zinzalias, Lucia Batzu, Gargi Mandal, Juliet Staunton, Frida Jernstedt, Kristina Rosqvist, Jonathan Timpka, Trinette van Vliet, Dhaval Trivedi, Aleksandra Podlewska, Miriam Parry, Daniel J. van Wamelen, Alexandra Rizos, Carolina Sportelli, Ana Laura Bonder, Guy Chung‐Faye, Cristian Falup‐Pecurariu, Simon Gaisford, Edoardo Moretto, Gwenaelle Le Gall, David Vauzour, Ana Rodriguez‐Mateos, Anna Sauerbier, Carmen Rodriguez Blazquez, Jonas Ghyselinck, Benoît Marsaux, Carmine Maria Pariante, Alessandra Borsini, Per Odin, Kallol Ray Chaudhuri

**Affiliations:** ^1^ Department of Basic and Clinical Neuroscience King's College London, Institute of Psychiatry, Psychology and Neuroscience London United Kingdom; ^2^ Parkinson's Foundation Center of Excellence at King's College Hospital London United Kingdom; ^3^ Department of Clinical Neurosciences Parkinson and Movement Disorders Unit, Fondazione IRCCS Istituto Neurologico “Carlo Besta” Milan Italy; ^4^ Psychology and Neuroscience, Department of Psychological Medicine Institute of Psychiatry, King's College London London United Kingdom; ^5^ Department of Neurology, Rehabilitation Medicine, Memory and Geriatrics Skåne University Hospital Lund Sweden; ^6^ Division of Neurology, Department of Clinical Sciences Lund University Lund Sweden; ^7^ Department of Neuroimaging King's College London; Institute of Psychiatry, Psychology and Neuroscience London United Kingdom; ^8^ Department of Neurology County Clinic Hospital Brasov Romania; ^9^ Faculty of Medicine Transilvania University Brasov Romania; ^10^ UCL School of Pharmacy University College London London United Kingdom; ^11^ Institute of Neuroscience, CNR Vedano al Lambro Italy; ^12^ Norwich Medical School University of East Anglia Norwich United Kingdom; ^13^ Department of Nutritional Sciences School of Life Course and Population Sciences, Faculty of Life Sciences and Medicine, King's College London London United Kingdom; ^14^ Faculty of Medicine and University Hospital Cologne, Department of Neurology University of Cologne Cologne Germany; ^15^ National Centre of Epidemiology and Network Centre for Biomedical Research in Neurodegenerative Diseases (CIBERNED) Carlos III Institute of Health Madrid Spain; ^16^ ProDigest BV Ghent Belgium; ^17^ Parkinson's Centre of Excellence at King's College Hospital London Dubai Hills United Arab Emirates; ^18^ Present address: Fondazione IRCCS Istituto Neurologico Carlo Besta, Department of Clinical Neurosciences, Parkinson and Movement Disorders Unit Milan Italy; ^19^ Present address: Parkinson's Center of Excellence at King's College Hospital London Dubai United Arab Emirates

**Keywords:** Parkinson's disease, gut microbiota, probiotics, inflammation, motor symptoms, non‐motor symptoms

## Abstract

**Background:**

Gut dysbiosis and gut‐brain‐axis involvement in people with Parkinson's disease (PwP) support the use of gut‐microbiota‐modulating interventions. Probiotics may help manage constipation in PwP; however, mechanisms underpinning additional beneficial properties are unknown.

**Objective:**

The aim was evaluating the effects of a probiotic (*Lacticaseibacillus rhamnosus, Lactobacillus acidophilus, Lactiplantibacillus plantarum* and *Enterococcus faecium)* on gut microbiota, inflammation, motor and non‐motor symptoms (NMS) in PwP and constipation.

**Methods:**

In this multicenter, randomized, double‐blind, placebo‐controlled trial (NCT05146921), PwP and constipation were randomized (1:1) to receive either the probiotic (4.08 × 10^8^ CFU/mL) or placebo orally (70 mL/day) for 12 weeks. The primary endpoint was the differential abundance of gut microbiota taxa between baseline and end‐of‐treatment in the active versus placebo group. Secondary/exploratory endpoints included changes in inflammatory cytokines plasma levels, short‐chain fatty acids (SCFAs) plasma and fecal levels, motor and NMS outcomes after 12 weeks. A per‐protocol analysis was performed.

**Results:**

Between July 17, 2019 and February 6, 2022, 74 participants were randomized. Data from 35 (probiotic) and 33 (placebo) participants were analyzed. Enrichments of bacteria with beneficial health‐related properties (*Odoribacteraceae*, *Enterococcaceae*, and *Blautia faecicola*) were observed in the active group compared to placebo (*P* ≤ 0.05). Proinflammatory cytokine TNF‐α plasma levels decreased with probiotic treatment and increased with placebo (*P* < 0.05). No changes in SCFAs levels were observed. Reductions in time‐to‐*on* and NMS scale scores (*P* < 0.05) were observed only in the active group.

**Conclusions:**

This probiotic was effective in beneficially enriching the gut microbiota with potential to reduce systemic inflammation, shortening time‐to‐*on* following levodopa administration, and alleviating NMS burden in PwP experiencing constipation. © 2025 The Author(s). *Movement Disorders* published by Wiley Periodicals LLC on behalf of International Parkinson and Movement Disorder Society. © 2025 The Author(s). *Movement Disorders* published by Wiley Periodicals LLC on behalf of International Parkinson and Movement Disorder Society.

Gastrointestinal dysfunction (GID) and pro‐inflammatory changes in the gut microbiota are increasingly regarded as integral aspects of Parkinson's disease (PD) and may have an early involvement in the disease, as also proposed in the body‐first subtype of PD.^1^, ^2^, ^3^ Gut microbiota studies showed pro‐inflammatory changes, including decreased abundance of short‐chain fatty acids (SCFAs)‐producing bacteria genera *Roseburia*,^4^, ^5^, ^6^, ^7^, ^8^, ^9^, ^10^, ^11^, ^12^ *Blautia*,^4^, ^6^, ^8^, ^9^, ^10^, ^11^, ^12^, ^13^ and *Faecalibacterium*,^4^, ^5^, ^6^, ^8^, ^14^, ^15^ as well as reduced fecal levels of these metabolites in people with PD (PwP) compared to controls.^9^, ^16^ SCFAs are known for their beneficial health‐related properties, such as enhancement of intestinal and blood–brain barriers function,^17^, ^18^, ^19^, ^20^, ^21^ and reduced expression of proinflammatory cytokines, including tumor necrosis factor α (TNF‐α).^22^ Of note, increased permeability of the intestinal and blood–brain barriers and elevated peripheral and central inflammatory levels were reported in PD.^23^ Furthermore, alterations in the abundance of specific bacterial taxa were associated with aspects of the PD stage and symptoms (motor and non‐motor symptoms [NMS]).^24^ As such, the use of gut‐microbiota‐modulating interventions (eg, probiotics, known to have anti‐inflammatory properties) is proposed as a possible therapeutic strategy for PD with potential beneficial effects on anxiety and memory deficits, in PD animal models.^25^ However, results from clinical studies are limited to level I evidence for the use of probiotics for constipation only in PD.^25^

Symprove is a probiotic suspension with an average bacterial population of 4.08 × 10^8^ CFU/mL and consisting of the following strains: *Lactobacillus acidophilus* NCIMB 30157, *Lactiplantibacillus plantarum* NCIMB 30173, *Lacticaseibacillus rhamnosus* NCIMB 30174, and *Enterococcus faecium* NCIMB 30176. Differently from most commercially available probiotics, Symprove is resistant to gastric acidity,^26^ one of the main physical and chemical barriers to a probiotic strain's viability. In vitro and in vivo data suggest intestinal, systemic, and central nervous system anti‐inflammatory effects of this probiotic in PD models.^27^, ^28^ Finally, this probiotic showed good tolerability and safety properties in patients with irritable bowel syndrome and inflammatory bowel diseases,^29^, ^30^ conditions frequently overlapping with PD.^31^, ^32^

Based on the above‐mentioned evidence, we hypothesized that the intake of this probiotic could change the gut microbiota and reduce levels of intestinal and systemic inflammation, potentially leading to motor and non‐motor effects, other than constipation in PwP. This hypothesis was tested by an exploratory randomized trial: the SymPD study. In this study, we aimed to assess the effects of Symprove oral intake on gut microbiota, markers of intestinal and systemic inflammation, as well as motor and NMS in PwP and constipation.

## Subjects and Methods

### Study Design

This was an exploratory 3‐month, randomized, double‐blind, placebo‐controlled, parallel‐arm, multicenter study run at the Parkinson's center of excellence at King's College Hospital (KCH) and King's College London, London, United Kingdom, and the Neurology Research Unit of Skåne University Hospital, Lund (LU), Sweden. The study was registered with ClinicalTrials.gov (NCT05146921) and followed the Consolidated Standards of Reporting Trials (CONSORT) reporting guidelines.^33^ It was adopted by the National Institute of Health Research (NIHR) in the United Kingdom and authorized by local ethics committees (details in Supplementary material).

### Participants

Consecutive PwP attending the Movement Disorders Clinics at KCH or LU were screened. Inclusion criteria were age ≥18 years; PD diagnosis according to the Movement Disorder Society criteria (MDS)^34^; functional constipation diagnosis according to the Rome IV criteria.^35^ Exclusion criteria included: device‐aided therapies use; history of inflammatory bowel disease or diseases of the intestine unrelated to PD (ie, celiac disease, etc.); previous gastrointestinal tract surgery; ongoing artificial nutrition; probiotics use over the last 12 months; previous intolerance and/or adverse reactions to probiotics; previous Symprove use; recent (within 4 weeks before the start of the study) or current use of any antibiotics; swallowing issues interfering with the safe intake of liquids; pregnancy or lactation; major active systemic diseases; any condition interfering with the ability to give informed consent; and enrolment in another simultaneous investigational trial. Dose and dosage of usual treatment for PD and/or comorbidity were maintained at a stable dose, whenever possible, during the intervention period. Changes in dose and dosage of laxatives (if required) were allowed for ethical reasons (probiotics are known to have beneficial effects on constipation).^36^ Participants were asked to maintain their diet and level of physical activity stable during the intervention period. Changes in medication regime, diet and physical activity were recorded through a structured interview. The use of antibiotics and/or other probiotics during the study period was regarded as a cause of early study termination.

### Randomization and Masking

Eligible patients were randomized to either the active or placebo arm with a 1:1 ratio using a computer‐generated randomization sequence by a research coordinator with no further involvement in the study (simple randomization). Allocation concealment was implemented by using coded sealed bottles. Participants and all study investigators were blinded to treatment allocation. Unblinding was done after the study database was locked. Active and placebo interventions were supplied by Symprove manufacturer: both looked identical in appearance, taste, weight, and packaging.

### Procedures

Participants were randomly allocated at entry to one of the two arms: active or placebo. Participants allocated to the active arm received 70 mL of Symprove once daily for 12 weeks. Participants allocated to the placebo arm received 70 mL of a liquid, which was identical to the active treatment in appearance, taste, weight, and packaging, but without any active ingredients. All participants were asked to keep the intervention refrigerated between 2°C and 7°C once the bottle was opened and to self‐administer 70 mL each morning on an empty stomach. Foods, fluids, and medications were allowed 30 minutes later. The dose of Symprove and the duration of treatment are safe and well‐tolerated according to previous studies in other medical conditions.^29^, ^30^, ^37^

Participants were invited to attend face‐to‐face study visits at baseline and 12 ± 2 weeks. Blood and fecal samples were collected at the two time points. Details on biological samples collection and processing are available in the Supplementary material. During the coronavirus disease 2019 (COVID‐19)‐related lockdown restrictions, study visits were performed virtually. Testing positive for COVID‐19 was regarded as a cause of early study termination, given the unknown effects of the infection on the study outcomes.

### Outcomes

The primary outcome measure was the differential abundance of gut microbiota taxa based on shallow shotgun sequencing between baseline and 12 weeks in the active versus placebo group. Secondary outcomes were changes between baseline and 12 weeks in the plasma levels of inflammatory cytokines such as TNF‐α, interferon (IFN)‐γ, interleukin (IL)‐6, IL‐8, and IL‐10 using the Human ProInflammatory Panel 1 Kit from Meso Scale Discovery. Exploratory outcomes were changes between baseline and 12 weeks in plasma and fecal levels of SCFAs via high‐performance liquid chromatography–tandem mass spectrometry, and changes in motor and NMS measured by the MDS Unified Parkinson's Disease Rating Scale (MDS‐UPDRS) part III (*on*) and part IV,^38^ duration of self‐reported “time‐to‐*on*” (time needed for the Parkinson medication to induce a motor and/or non‐motor improvement),^39^ and NMS scale (NMSS).^40^ Safety, tolerability, and compliance data (through a structured interview) were gathered during the study.

### Sample Size

This was an exploratory study, as no prior trials had examined the effects of this probiotic on gut microbiota in PwP. Consequently, a formal sample size calculation based on prior effect estimates was not feasible. However, assuming a low‐probability event occurring in 5% of the population, a sample size of 59 participants would provide a 95% probability of observing at least one such event, supporting the study's capacity to detect rare outcomes.^41^ This sample size is, in fact, appropriate for detecting small effect sizes in pilot studies.^42^ To account for an anticipated attrition rate of up to 25%, the target sample size was increased to 74 participants.

## Statistical Analysis

### Study Population

First, between‐group differences of baseline socio‐demographics, PD‐related data (disease duration, Hoehn and Yahr stage), medication for PD, motor symptoms (MDS‐UPDRS part III and IV), NMS (NMSS), body mass index (BMI), smoking status, nutrition and physical activity‐related data (measured by the nutrition and physical activity questionnaire), and constipation‐related data were analyzed using Independent‐samples *t* test, Mann–Whitney U test, Pearson χ^2^ test, Fisher's exact test, where appropriate.

Subsequently, a longitudinal analysis to evaluate changes in PD medication, nutrition, and physical activity‐related data to check participants' compliance with study instructions (patients were asked to maintain stable PD medication, nutritional, and physical activity habits throughout the study) was performed. Within‐group changes were assessed using marginal homogeneity test or Wilcoxon signed‐rank test, where appropriate. Finally, descriptives of compliance and safety‐related data were reported.

### Gut Microbiota

α‐Diversity, β‐diversity, and differential abundance analyses were performed. Detailed information on gut microbiota analysis is available in the Supplementary material (including Figs S1–S3).

### Inflammatory Markers

A longitudinal analysis to evaluate within‐group changes in the plasma levels of inflammatory cytokines such as IFN‐γ, TNF‐α, IL‐6, IL‐8, and IL‐10 was performed using Wilcoxon signed‐rank test or paired *t* test, where appropriate. In addition, between‐group differences of normalized changes (Δ*N* = [follow‐up value − baseline value]/baseline value) were analyzed using Mann–Whitney T test or Independent‐samples *t* test, where appropriate. A similar approach was used for fecal and plasma levels of SCFAs.

### Motor and NMS

A longitudinal analysis to evaluate within‐group changes in the severity of motor symptoms (MDS‐UPDRS part III and IV, and time‐to‐*on*), and NMS (NMSS total and domains scores) was performed using Wilcoxon signed‐rank test or paired *t* test where appropriate. Effect size for the above‐mentioned within‐group changes was measured and expressed as Cohen's *d* for continuous variables or Cohen's g for dichotomous variables.

Statistical analyses were performed using R version 4.2.1, Rstudio version 2022.02.3 + 492 and Statistical Package for the Social Sciences (SPSS), version 28.0.0 (IBM, Armonk, NY). A *P*‐value of ≤ 0.05 was considered statistically significant. As this was an exploratory study primarily investigating the underlying biological mechanisms of the intervention, correction for multiple comparisons was not applied,^43^ and a per‐protocol analysis was performed.^44^

### Data Sharing

The datasets used and/or analyzed during the current study are available from the corresponding authors and study sponsors on reasonable request.

## Results

### Study Population

**FIG. 1:**
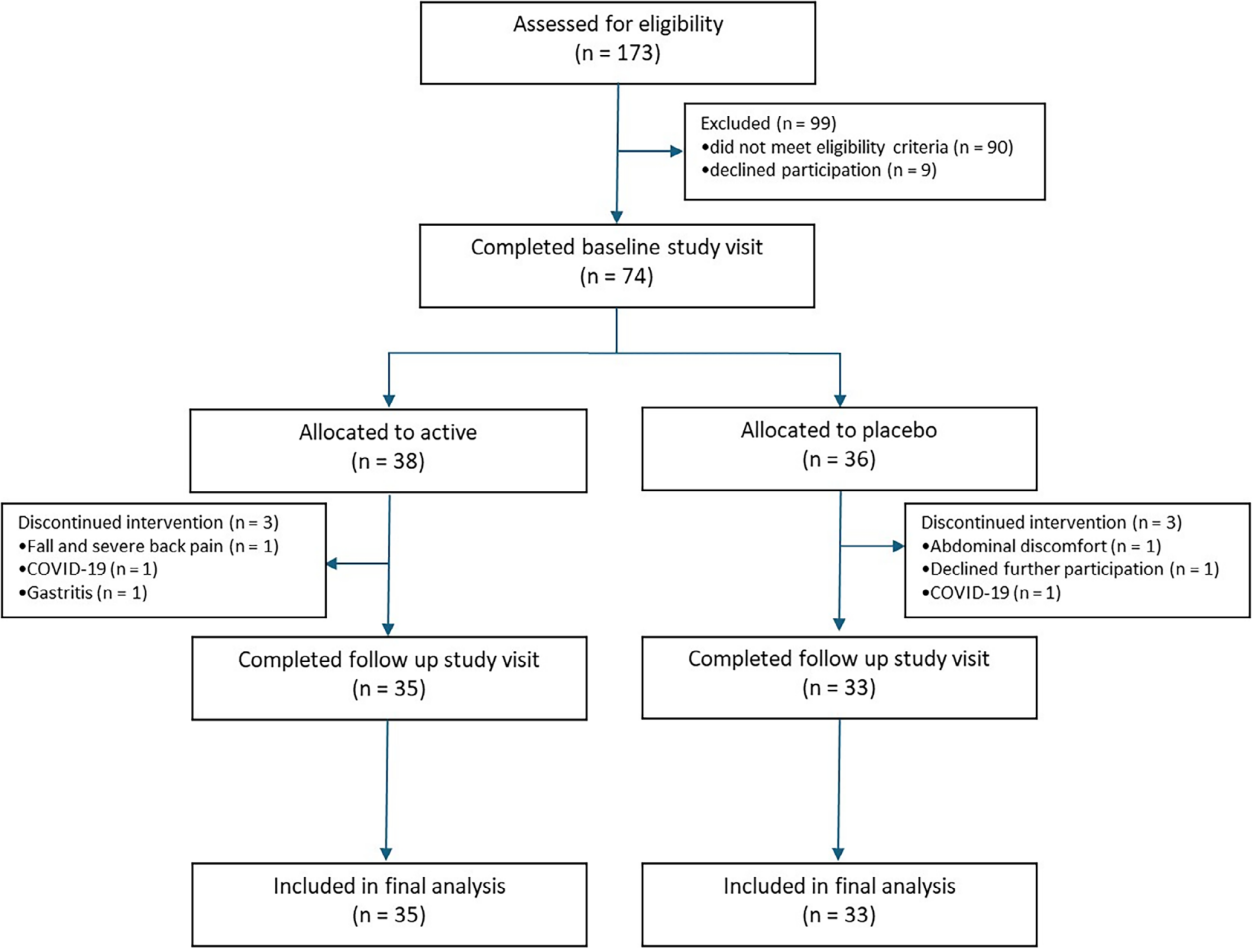
Consolidated Standards of Reporting Trials (CONSORT) diagram of the SymPD study. COVID‐19, coronavirus disease 2019; n, number. [Color figure can be viewed at wileyonlinelibrary.com]

Recruitment was performed between July 17, 2019 and February 6, 2022. Of 173 patients screened, 74 met the eligibility criteria and were randomized to either the active (n = 38) or placebo (n = 36) group. The retention rate was high (92%), with six of 74 participants discontinuing the intervention. Reasons for discontinuation are provided in Fig 1 and were mostly not related to the intervention.

Socio‐demographics, PD‐related data including disease duration, medication, and the severity of motor and NMS, nutrition, physical activity, and smoking status as well as constipation‐related features were balanced between groups at baseline (Tables 1 and S1).

**TABLE 1:** Baseline socio‐demographics, PD, BMI, smoking status, and constipation‐related data

	Active (n = 35)	Placebo (n = 33)	*P*
Age at assessment (y)	68.17 ± 8.29	65.24 ± 7.73	0.137^a^
Sex assigned at birth (male, (%))	23 (65.7)	24 (72.7)	0.532^b^
Ethnicity			0.493^b^
White (%)	33 (94.3)	32 (97.0)	
Asian (%)	2 (5.7)	0 (0.0)	
Black African (%)	0 (0.0)	1 (3.0)	
Disease duration (y)	7.60 ± 5.87	8.48 ± 5.99	0.522^c^
H&Y stage			0.566^b^
2 (%)	19 (54.3)	21 (63.6)	
3 (%)	13 (37.1)	8 (24.2)	
4 (%)	3 (8.6)	4 (12.1)	
Medication for PD			
LEDD (mg/day)	706.64 ± 477.89	717.56 ± 449.53	0.801^c^
Levodopa (%)	33 (94.3)	30 (90.9)	0.668^b^
Dopamine agonists (%)	19 (54.3)	20 (60.6)	0.598^b^
COMT‐I (%)	10 (28.6)	8 (24.2)	0.686^b^
MAOB‐I (%)	12 (34.3)	19 (57.6)	0.054^b^
*On*‐MDS‐UPDRS part III	35.49 ± 17.59	35.85 ± 16.15	0.830^c^
MDS‐UPDRS part IV	3.86 ± 3.71	3.82 ± 3.98	0.861^c^
NMSS	70.71 ± 45.22	56.88 ± 30.43	0.308^c^
BMI (kg/m^2^)	25.76 ± 4.15	25.90 ± 3.98	0.892^a^
Smoking status			
Smoker (%)	1 (2.9)	2 (6.1)	0.608^b^
Non‐smoker (%)	34 (97.1)	31 (93.9)	0.608^b^
Ex‐smoker (%)	15 (42.9)	14 (42.4)	0.971^b^
Constipation			
Frequency of any bowel movements per week	4.26 ± 1.97	4.58 ± 2.96	0.915^c^
Taking laxative (%)	21 (60.0)	18 (54.5)	0.649^b^
Frequency of laxatives per week	2.97 ± 3.58	3.23 ± 4.20	0.897^c^

*Note*: Data presented as mean ± standard deviation or number (percentage). Differences between groups were tested using independent‐samples t test or Pearson χ2 test or Fisher's exact test or Mann‐Witney U test as appropriate. Two‐sided values of *P ≤*  0.05 were considered statistically significant (in **bold**). LEDD was calculated according to the conversion formulae proposed by Tomlinson et al.^45^

Abbreviations: BMI, body mass index; y, years; H&Y, Hoehn and Yahr scale; LEDD, levodopa equivalent daily dose; COMT‐I, catechol‐O‐methyltransferase inhibitors; MAOB‐I, Monoamine oxidase B inhibitors; MDS‐UPDRS, Movement Disorder Society Unified Parkinson's Disease Rating Scale; NMSS, Non‐Motor Symptoms Scale; PD, Parkinson's disease.

^a^ Independent‐samples *t* test.

^b^ Pearson χ^2^ test or Fisher's exact test.

^c^ Mann‐Witney *U* test.

No statistically significant changes in PD medication (levodopa equivalent daily dose [LEDD]), nutrition and physical activity‐related data were observed between baseline and follow‐up in both the active and the placebo groups (Table S2).

Concerning compliance with the intervention, 44 (65%) participants took the intervention as per instructions (once daily), 21 (31%) missed one dose per week, and three (4%) missed more than one dose per week. The active treatment was well tolerated. The same number of adverse events (n = 11) and a similar number of withdrawals because of adverse events (n = 3 [8%] and n = 2 [6%]) were reported in the active and placebo group, respectively (Table S3). No serious adverse events were reported during the study period.

### Gut Microbiota

Of 68 participants who completed the study, baseline and follow‐up stool samples from 58 participants (30 from the active and 28 from the placebo group) were analyzed, as 10 participants did not collect stool samples following study instructions (ie, insufficient sample).

α‐Diversity and β‐diversity metrics did not differ between groups at baseline, and no significant changes were detected between groups after the intervention period when accounting for natural temporal variation using the placebo group as reference (Figs S4 and S5).

**FIG. 2:**
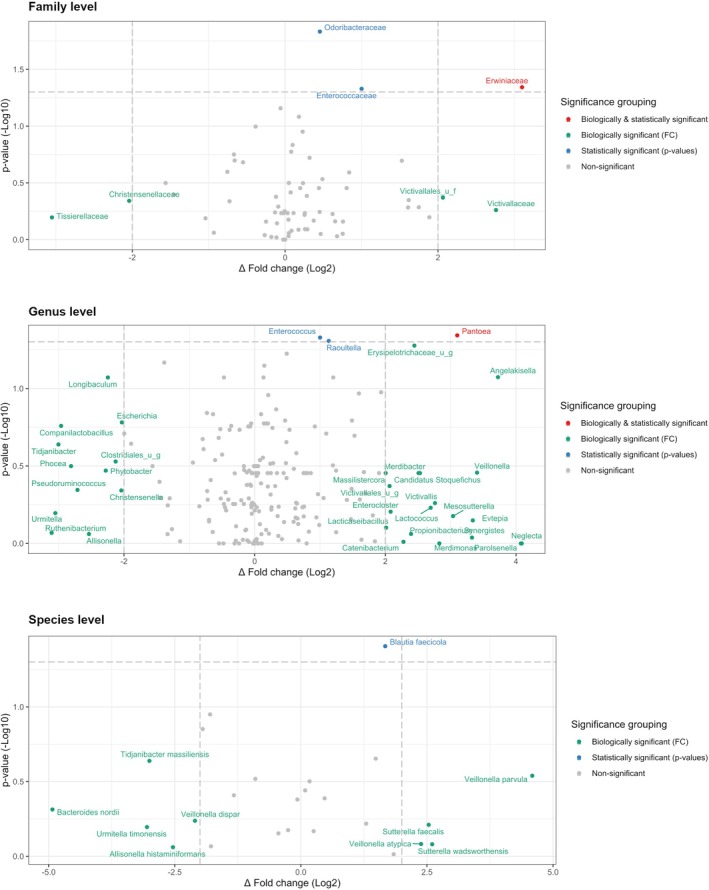
Differential abundance analysis showing differences in community composition between microbial populations in active and placebo groups (top family level, middle genus level, bottom species level—top 25 most abundant taxa). The obtained scatter plots classify bacterial taxa into four different categories based on their abundance in compared groups: (A) not significant and not biologically relevant (gray), (B) biologically relevant, but not statistically significant (green), (C) statistically significant, but not biologically relevant (blue), and (D) biologically and statistically significant (red). Positive fold changes indicate enrichment in active group (right‐hand side), negative fold changes indicate enrichment in the placebo group (left‐hand side). FC, fold changes. [Color figure can be viewed at wileyonlinelibrary.com]

Differential abundance analysis showed that the active treatment was associated with the biologically and statistically significant enrichment of the *Erwiniaceae* family (Δ Log2 = 3.10, *P* = 0.05), which was driven by the biologically and statistically significant enrichment of the *Pantoea* genus (Δ Log2 = 3.10, *P* = 0.05). Other statistically significant enrichments included the *Odoribacteraceae* (Δ Log2 = 0.45, *P* = 0.01) and *Enterococcaceae* (Δ Log2 = 1.00, *P* = 0.05) families (the latter driven by the *Enterococcus* genus [Δ Log2 = 1.00, *P* = 0.05], including one of the probiotic agents of the active treatment [*Enterococcus faecium*]), and the species *Blautia faecicola* (Δ Log2 = 1.67, *P* = 0.04) (Fig. 2).

### Inflammatory Markers

Blood samples from 22 and 19 participants from the active and placebo groups, respectively, were collected and analyzed (blood samples were not available for all study participants because of the COVID‐19 pandemic temporary restrictions and the virtual nature of the assessments performed during that period). Statistically significant reductions in the plasma levels of IL‐6 (*P* = 0.028) and TNF‐α (*P* = 0.024) were observed in the active treatment group, whereas a significant increase in the plasma levels of IL‐6 (*P* = 0.040) and TNF‐α (*P* = 0.005) was observed in the placebo group (Table 2). Analyses of between‐group differences of normalized changes (Δc/c_0_ = [follow‐up value – baseline value]/baseline value) confirmed a statistically significant difference in the plasma levels of TNF‐α between the active and placebo groups (*P* < 0.001) (Fig. S6). No statistically significant changes were observed in fecal and plasma levels of SCFAs in the two treatment groups (data not shown).

**TABLE 2:** Changes in plasma levels of inflammatory cytokines in the active and placebo groups

Cytokine (pg/mL)	Active (n = 22)			Placebo (n = 19)		
	Baseline	Follow‐up	*P*	Baseline	Follow‐up	*P*
IFN‐γ	6.60 ± 4.19	6.09 ± 5.00	0.372	3.90 ± 2.30	12.93 ± 41.58	0.778
TNF‐α	2.04 ± 1.40	1.69 ± 0.93	**0.024**	1.37 ± 0.42	1.69 ± 0.63	**0.005**
IL‐6	1.01 ± 0.49	0.81 ± 0.45	**0.028**	1.16 ± 1.23	1.45 ± 2.34	**0.040**
IL‐8	13.00 ± 4.37	11.69 ± 3.91	0.149	11.49 ± 5.23	11.17 ± 3.97	0.520
IL‐10	0.33 ± 0.34	0.32 ± 0.49	0.115	0.19 ± 0.09	0.19 ± 0.08	0.809

*Note*: Data presented as mean ± standard deviation. Data were analyzed using the Wilcoxon signed‐rank test. Two‐sided values of *P ≤*  0.05 were considered statistically significant (in **bold**).

Abbreviations: IFN‐γ, interferon γ; TNF‐α, tumor necrosis factor‐α; IL‐6, interleukin 6; IL‐8 interleukin 8; IL‐10, interleukin 10.

### Motor and NMS

Data from 35 and 33 participants from the active and placebo groups, respectively, were analyzed. In relation to motor outcomes, a statistically significant reduction of time‐to‐*on* was observed in the active group between baseline and follow‐up (*P* = 0.027, Cohen's *d* = 0.709). No other statistically significant within‐group changes in motor outcomes were observed in either the active or the placebo group (Table 3).

**TABLE 3:** Changes in motor and non‐motor outcomes in the active and placebo groups

	Active (n = 35)				Placebo (n = 33)			
	Baseline	Follow‐up	*P*	*d*	Baseline	Follow‐up	*P*	*d*
*On*‐MDS‐UPDRS‐III	35.49 ± 17.59	30.88 ± 16.76	0.149	0.366	35.85 ± 16.15	30.60 ± 14.99	0.058	0.505
MDS‐UPDRS‐IV	3.86 ± 3.71	4.54 ± 4.05	0.133	0.440	3.82 ± 3.98	3.42 ± 3.00	0.740	0.082
Time‐to‐*on* (min)	31.43 ± 25.22	23.95 ± 27.50	**0.027**	0.709	32.70 ± 38.31	27.65 ± 28.74	0.260	0.362
NMSS	70.71 ± 45.22	61.34 ± 47.20	**0.005**	0.704	56.88 ± 30.43	54.36 ± 36.29	0.440	0.191

*Note*: Data presented as mean ± standard deviation. Within‐group changes were tested using Wilcoxon‐signed‐rank test. Two‐sided values of *P* ≤ 0.05 were considered statistically significant (in **bold**). Effect size was expressed as Cohen's *d*.

Abbreviations: MDS‐UPDRS, Movement Disorder Society Unified Parkinson's Disease Rating Scale; NMSS, Non‐Motor Symptoms Scale.

In relation to non‐motor outcomes, a statistically significant reduction in NMSS score was observed in the active group (*P* = 0.005, Cohen's *d* = 0.704) (Table 3), which was driven by statistically significant reductions in the sleep/fatigue (*P* = 0.007, Cohen's *d* = 0.687) and gastrointestinal (*P* < 0.001, Cohen's *d* = 0.926) domains scores and more specifically by the fatigue (*P* = 0.025, Cohen's *d* = 0.554) and constipation (*P* = 0.003, Cohen's *d* = 0.774) items, respectively (data not shown).

## Discussion

To the best of our knowledge, this international, multicenter, randomized, double‐blind, placebo‐controlled trial is the first study suggesting beneficial effects of this probiotic, Symprove, on gut microbiota, and potentially on markers of systemic inflammation, aspects of motor and NMS, other than constipation, in PwP and GID. Specifically, 12‐week Symprove intake was associated with the enrichment of bacteria with beneficial health‐related properties (families *Odoribacteraceae*, *Enterococcaceae*, and species *Blautia faecicola*), reductions in the plasma levels of the proinflammatory marker TNF‐α, time‐to‐*on* after levodopa intake, and overall NMS burden driven by improvements in the sleep/fatigue and gastrointestinal domains, and more specifically in the fatigue and constipation items, respectively, of the NMSS. These findings are of interest and shed light on the further development of probiotics as a potential treatment for PD.

Regarding the gut microbiota, α‐ and β‐diversity metrics did not differ between active and placebo groups before intervention, indicating successful randomization and comparable microbiota profiles. Furthermore, no significant changes in α‐ and β‐diversity metrics were detected between groups after the intervention period. Such stability is favorable for a targeted intervention, which seeks to influence specific taxa or functions without broadly altering overall diversity, thereby reducing the risk of inducing dysbiosis. Differential abundance analysis revealed significant changes in the gut microbiota even after taking into consideration the time and placebo effects.^46^ Many of the significantly enriched bacterial taxa associated with the active treatment (*Odoribacteraceae*, *Enterococcaceae*, and *Blautia faecicola*) have been linked with gut health. The family *Odoribacteraceae* is known for its protective effects against harmful bacteria, inherent to its capacity to convert primary bile acids into secondary bile acids,^47^ such as deoxycholic acid, lithocholic acid (LCA), and ursodeoxycholic acid. Sato and colleagues^47^ proposed that elevated fecal levels of LCA, and specifically of its isoform isoallo‐LCA produced by *Odoribacteraceae*, represent a biomarker for healthy ageing. Moreover, evidence from in vitro and in vivo studies suggests that bile acids such as LCA can modulate inflammation by inhibiting nod‐like receptor family pyrin domain containing 3 (NLRP3) inflammasome activation,^48^ which contributes to α‐synuclein aggregation.^49^ The enrichment of *Enterococcaceae* with the active treatment was driven by *Enterococcus genus* and potentially by one of the probiotic agents, *Enterococcus faecium* 30176, which belongs to Clade B according to the European Food Safety Authority and is safe to use. *Enterococcus faecium* survives low pH values and bile acids, and being a commensal bacterium, it can hamper the growth of harmful bacteria such as *Salmonella serovars*, *Shigella spp*., and *Enterobacter spp*.^50^ Treatment with the probiotic was also associated with enrichment of the SCFAs‐producer *Blautia faecicola* whose abundance is known to be reduced in PwP compared to controls.^1^, ^51^ In a recent ex vivo study, where stool samples from PwP were left to ferment in an in vitro model simulating the human gastrointestinal tract (M‐SHIME), increased levels of SCFAs were measured after Symprove administration.^27^ Furthermore, increased fecal levels of SCFAs and decreased plasma levels of pro‐inflammatory markers (lipopolysaccharide, TNF‐α, IL‐1β, and IL‐6) were observed in a mouse model of PD after 24‐day Symprove supplementation.^28^ In the SymPD study, we observed a significant difference in the normalized changes of plasma TNF‐α levels between the active and placebo groups, with probiotic treatment associated with a reduction in TNF‐α, while levels increased in the placebo group. This finding is consistent with evidence in elderly populations linking constipation to elevated systemic levels of pro‐inflammatory cytokines such as IL‐6 and TNF‐α, supporting the notion of constipation as a contributor to chronic low‐grade inflammation.^52^ Given that probiotics have demonstrated efficacy in alleviating constipation in PD^36^—and our study corroborated this finding—it is plausible that the reduction in cytokine levels observed in the probiotic group reflects an amelioration of constipation‐associated inflammation. Conversely, the increase in cytokine levels in the placebo group may indicate persistent or exacerbated constipation‐related inflammation. Nonetheless, we emphasize cautious interpretation of these results because of the limited sample size and exploratory nature of the study. No changes in fecal and plasma levels of SCFAs were detected in the study. This may be attributed to the high volatility and hydrophilic nature of SCFAs, which complicate their accurate measurement, combined with a sample size that was not specifically powered for this outcome.

In relation to motor symptoms, the active treatment was associated with a reduction of time‐to‐*on* related to levodopa intake. This is the first controlled study using probiotics to suggest such clinically relevant motor benefits. A possible mechanism underpinning this observation could be the consolidated beneficial effect of probiotics on slow‐transit constipation, which is a barrier to levodopa transport and, therefore, absorption.^53^ This probiotic was also associated with a reduction of the NMS total burden, which negatively affects quality of life in PD.^54^ This finding was driven by a reduction of the gastrointestinal domain score (and specifically the constipation item score) and of the sleep/fatigue domain score (and specifically the fatigue item score) of the NMSS. Several trials have already shown that probiotics can improve constipation‐related outcomes in PwP and are recommended by the Evidence‐Based Medicine Review by the MDS.^25^, ^36^ Results from the SymPD study further support this observation. Candidate mechanisms include: (1) the observed increased abundance of *Odoribacteraceae* and increased levels of secondary bile acids, which can act as natural laxatives^55^, ^56^, ^57^, ^58^; (2) *Lacticaseibacillus casei*, *Lactobacillus acidophilus*, and *Lactiplantibacillus plantarum*, which are contained in Symprove, might stimulate mucin secretion,^59^, ^60^ which serves as a lubricant and facilitate stool passage.^61^ The potential beneficial effect of Symprove on fatigue could be underpinned by the anti‐inflammatory properties of the active agent.^28^ Of note, supplementation of probiotics other than Symprove has already been associated with improvements in fatigue‐related outcomes in patients with chronic fatigue syndrome,^62^ and post‐COVID‐19 fatigue.^63^

The probiotic was well tolerated in PwP and constipation as demonstrated by the same adverse events number recorded for both study groups, the absence of serious adverse events, and an overall high study retention rate (92%), also in agreement with previous studies.^29^, ^30^

In relation to the study cohort, the treatment groups were balanced for socio‐demographics, PD‐related data (PD medication and severity of motor as well as NMS), BMI, nutrition, physical activity, smoking status as well as constipation‐related features at baseline. Although antiparkinsonian medications such as levodopa and COMT inhibitors can influence gut microbiota composition, the randomization process resulted in a balanced distribution of these medications between groups, reducing potential bias.^11^ In addition, no significant changes in LEDD, smoking‐status, nutrition, and physical activity‐related data during the treatment period were observed in either group. These observations, in combination with the multicenter international setting and the use of broad eligibility criteria from a real‐world outpatient clinic setting, contribute to the internal and external validity of the study.^64^

Some limitations need to be acknowledged, such as the lack of detailed nutritional, physical‐activity, gastrointestinal, and compliance evaluations. Additional limitations include the two‐step transport of stool samples (from patient home to study center and from study center to central laboratory), and to reduce the possible impact of transport on gut microbiota, the samples were asked to be immediately frozen at −20°C at patients home and were transported without breaking the cold chain. In addition, all samples from both centers were analyzed at the same time by the same central laboratory to ensure consistent analysis and results. Corrections for multiple comparisons were not applied, and an intention‐to‐treat analysis was not performed, and instead, a per‐protocol approach was adopted. These decisions were driven by the exploratory nature of the study, which was designed primarily to investigate the underlying biological mechanisms of the intervention.^43^, ^44^ Finally, because of the temporary COVID‐19 pandemic‐related restrictions, blood sampling was not possible for all participants. Moreover, the pandemic might have influenced the study outcomes, given possible deleterious direct and indirect effects of COVID‐19 on the symptoms of PwP.^65^, ^66^ The lack of diversity in ethnic groups, with more than 90% of the population recruited being white Caucasian, is a concern, as highlighted in recent studies.^67^ This issue, as well as more granular monitoring of motor and non‐motor states using objective outcome measures, a longer intervention period, and a larger sample size, may be considered for future trials.^68^

## Conclusions

Results from the SymPD study suggest that a 12‐week intake of the probiotic Symprove was effective in beneficially enriching the gut microbiota with potential to reduce systemic inflammation, time‐to‐*on* as well as total NMS burden (driven by improvements of constipation and fatigue) in PwP and constipation. As gut health is an integral part of the clinical management of PD,^69^ our findings highlight the need for further investigation into probiotics, such as Symprove, as potential therapeutic strategies in PwP.

## Author Roles

(1) Research project: A. Conception, B. Organization, C. Execution; (2) Statistical Analysis: A. Design, B. Execution, C. Review and Critique; (3) Manuscript: A. Writing of the First Draft, B. Review and Critique.

V.L.: 1B, 1C, 2A, 2B, 2C, 3A, 3B

P.Z.: 1B, 1C, 3B

L.B.: 2A, 2B, 2C, 3B

G.M.: 1C, 3B

J.S.: 1C, 3B

F.J.: 1B, 1C, 3B

K.R.: 1B, 1C, 3B

JT: 1B, 1C, 3B

T.v.V.: 1B, 3B

D.T.: 1B, 3B

A.P.: 1B, 3B

M.P.: 1C, 3B

D.J.v.W.: 2C, 3B

A.R.: 1B, 3B

C.S.: 1B, 1C, 3B

A.L.B.: 1C, 3B

G.C.F.: 1B, 3B

C.F.P.: 1B, 3B

S.G.: 1B, 3B

E.M.: 2B, 2C, 3B

G.L.G.: 1C, 3B

D.V.: 1C, 2C, 3C

A.R.M.: 1C, 2C, 3C

A.S.: 1B, 3B

C.R.B.: 2C, 3B

J.G.: 1C, 2B, 3B

B.M.: 1C, 2B, 2C, 3B

C.M.P.: 1B, 3B

A.B.: 1B, 3B

P.O.: 1B, 3B

K.R.C.: 1A, 1B, 2C, 3B

All authors had full access to all the data in the study and had final responsibility for the decision to submit for publication.

## Financial Disclosures

V.L. has received lecture fees from Bial, Everpharma, and Zambon outside of the submitted work. C.F.P. reports editor fees from Springer and Elsevier, speaker fees from AbbVie, Zentiva and from the International Parkinson and MDS, outside of the present work. P.O. has received lecture fees and/or advisory board fees from AbbVie, Bial, Britannia, NordicInfu Care, Stada, and Zambon. K.R.C. received honoraria or consultation fees from UCB, AbbVie, US WorldMeds, Otsuka, and Britannia, outside the submitted work. The remaining authors declare no conflicts of interest.

## Supporting information


**Figure S1.** Example of output generated with volcano plots. Statistical significance was plotted in function of fold change, classifying bacterial taxa into four categories: not significant and not biologically relevant (grey), biologically relevant but not statistically significant (green), statistically significant, but not biologically relevant (blue), and biologically and statistically significant (red).
**Figure S2.** Schematic representation of the approach followed to assess active treatment effects. Per participant and for each bacterial taxa (S1‐Sx), fold changes (FC) were calculated (ratio of abundance at follow‐up (T1) versus baseline (T0) (FC(T1/T0)) for the active and placebo groups. Then, bacterial taxa were identified for which the fold change was different in the active group from the placebo group.
**Figure S3.** Equation 1: Calculation of differences in fold change between active and placebo groups for a given bacterial taxon. Abbreviations: T0 = baseline, T1 = follow‐up.
**Table S1.** Baseline nutrition and physical exercise‐related data.
**Table S2.** Evaluation of changes in Parkinson's medication, nutrition, and physical activity‐related data.
**Table S3.** Adverse events and related withdrawals.
**Figure S4.** Gut microbiota α‐ and β‐diversity at the species level show no differences between groups at either time point. (A) Principal coordinates analysis (Bray–Curtis dissimilarity) of species‐level relative microbiota profiles. PERMANOVA performed separately for each time point showed no significant differences between Active and Placebo groups at T0 (*P* = 0.400) or T1 (*P* = 0.579). (B) α‐diversity metrics, including Observed richness (T0: *P* = 0.127; T1: *P* = 0.191), Shannon diversity (T0: *P* = 0.165; T1: *P* = 0.501), Inverse Simpson diversity (T0: *P* = 0.242; T1: *P* = 0.583), and Pielou's evenness (T0: *P* = 0.694; T1: *P* = 0.969), did not differ significantly between groups at either time point. Abbreviations: ns = non‐significant, T0 = baseline; T1 = follow‐up.
**Figure S5.** Gut microbiota α‐ and β‐diversity at the species level show no microbial shifts. (A) Principal coordinates analysis (Bray–Curtis dissimilarity) of species‐level relative microbiota profiles with 95% confidence ellipses. PERMANOVA indicated no significant effects of Group (Active vs. Placebo; *P* = 1.000), Time (T0 vs. T1; *P* = 0.353), or their interaction (*P* = 0.514). Beta‐dispersion analysis showed no differences in inter‐individual variability across Group (*P* = 0.254), Time (*P* = 0.869), or Group × Time (*P* = 0.594), indicating that β‐diversity patterns were not driven by changes in within‐group heterogeneity. (B) Pairwise comparisons of distance to centroid showed no changes from T0 to T1 within groups (Placebo: *P* = 0.764; Active: *P* = 0.553) and no differences between groups at either time point (T0: *P* = 0.180; T1: *P* = 0.573), confirming stable within‐group variability over time and between treatments. (C) Within‐subject Bray–Curtis dissimilarity between T0 and T1 did not differ between groups (*P* = 0.562), although values were greater than zero within both Placebo (*P* < 0.001) and Active (*P* < 0.001) groups, suggesting natural temporal variability unrelated to treatment. (D) Δα‐diversity (T1 – T0) for Observed richness, Shannon diversity, Inverse Simpson diversity, and Pielou's evenness showed no significant differences between groups and no within‐group deviations from baseline. Abbreviations: ns = non‐significant, T0 = baseline; T1 = follow‐up.
**Figure S6.** Between‐group differences in normalised changes (Δc/c_0_) of plasma levels of inflammatory cytokines. Plasma levels of IFN‐γ, TNF‐α, IL‐6, IL‐8, and IL‐10 were analysed using the Human ProInflammatory Panel 1 Kit from Meso Scale Discovery Mesoscale Discovery (MSD). Normalised delta = ((follow up value – baseline value)/ baseline value). Data presented as median and interquartile range. Data were analysed using the Mann–Whitney T test. *** *P* < 0.001. Abbreviations: IFNγ: interferon gamma; IL6: interleukin 6; IL8 interleukin 8; IL10: interleukin 10; TNFα: Tumor necrosis factor‐α.

## Data Availability

The data that support the findings of this study are available on request from the corresponding authors and study sponsors. The data are not publicly available due to privacy or ethical restrictions.
