# Supplementary material for: Effects of a Four‐Strain Probiotic on Gut Microbiota, Inflammation, and Symptoms in Parkinson's Disease: A Randomized Clinical Trial
Source: Mov Disord. 2025 Oct 23;40(12):2710–21. doi: 10.1002/mds.70047 (PMC12710184; doi:10.1002/mds.70047)
Supplement: Supplementary file 1 — Figure S1. Example of output generated with volcano plots. Statistical significance was plotted in function of fold change, classifying bacterial taxa into four categories: not significant and not biologically relevant (grey), biologically relevant but not statistically significant (green), statistically significant, but not biologically relevant (blue), and biologically and statistically significant (red). Figure S2. Schematic representation of the approach followed to assess active treatment effects. Per participant and for each bacterial taxa (S1‐Sx), fold changes (FC) were calculated (ratio of abundance at follow‐up (T1) versus baseline (T0) (FC(T1/T0)) for the active and placebo groups. Then, bacterial taxa were identified for which the fold change was different in the active group from the placebo group. Figure S3. Equation 1: Calculation of differences in fold change between active and placebo groups for a given bacterial taxon. Abbreviations: T0 = baseline, T1 = follow‐up. Table S1. Baseline nutrition and physical exercise‐related data. Table S2. Evaluation of changes in Parkinson's medication, nutrition, and physical activity‐related data. Table S3. Adverse events and related withdrawals. Figure S4. Gut microbiota α‐ and β‐diversity at the species level show no differences between groups at either time point. (A) Principal coordinates analysis (Bray–Curtis dissimilarity) of species‐level relative microbiota profiles. PERMANOVA performed separately for each time point showed no significant differences between Active and Placebo groups at T0 (P = 0.400) or T1 (P = 0.579). (B) α‐diversity metrics, including Observed richness (T0: P = 0.127; T1: P = 0.191), Shannon diversity (T0: P = 0.165; T1: P = 0.501), Inverse Simpson diversity (T0: P = 0.242; T1: P = 0.583), and Pielou's evenness (T0: P = 0.694; T1: P = 0.969), did not differ significantly between groups at either time point. Abbreviations: ns = non‐significant, T0 = baseline; T1 = follow‐up. Figure S5. Gu [file MDS-40-2710-s001.docx]

**SUPPLEMENTARY MATERIAL**

**SUPPLEMENTARY METHODS**

**Local ethics committees**

UK Research Ethics Committees reference 19/LO/0319 for KCH and Swedish Ethical Review Authority references 2019-05081, 2020-01331 and 2022-01153-02 for LU. All patients gave written informed consent prior to study procedures in accordance with the Declaration of Helsinki.

**Stool samples collection, storage, transport, and processing (Shallow shotgun sequencing)**

Faecal samples were frozen immediately at -20°C at patients' homes after collection and transported to the central laboratory without breaking the cold chain. DNA was extracted from the faecal samples using the Cetyl trimethylammonium bromide method.(1) DNA libraries were prepared using the Nextera XT DNA Library Preparation Kit (Illumina) and IDT Unique Dual Indexes with total DNA input of 1 ng. Genomic DNA was fragmented using a proportional amount of Illumina Nextera XT fragmentation enzyme. Unique dual indexes were added to each sample, followed by 12 cycles of PCR to construct libraries. DNA libraries were purified using AMpure magnetic Beads (Beckman Coulter) and eluted in QIAGEN EB buffer. DNA libraries were quantified using Qubit 4 fluorometer and Qubit™ dsDNA HS Assay Kit. Libraries were then sequenced on Illumina NovaSeq platform 2x 150 bp. Unassembled sequencing reads were directly analysed as described elsewhere for multi-kingdom microbiota analysis and quantification of organisms' relative abundances.(2-5)

**Blood samples collection and processing**

Blood samples were collected during the study visits in the morning at the same time for each patient and immediately frozen at -20°C before processing. Detailed information on the cytokines analysis procedure can be found in previous publications.(6)

**Gut microbiota α- and β-diversity analyses**

Community composition at baseline (T0) and follow-up (T1) were analysed. Rare taxa were removed prior to α- and β-diversity analyses to minimise the influence of sequencing artefacts and low-prevalence species on diversity estimates and community dissimilarities. Briefly, data were provided as total sum scaled (TSS) normalised counts and processed using the phyloseq package (v1.52.0). Species present in less than 5% of samples and with a mean relative abundance lower than 0.01% across all samples were removed, resulting in the exclusion of 229 and 44 taxa, respectively. These filtering steps reduced the dataset from 533 to 273 taxa, corresponding to 1.76 ± 2.62% of total counts considered as background noise (false positives or rare taxa). All excluded taxa were aggregated into a composite “Others” category to preserve total relative abundance.

Alpha-diversity was assessed at the species level by calculating Observed richness, Shannon diversity, Inverse Simpson diversity, and Pielou’s evenness using the estimate_richness() function from phyloseq. Between-group comparisons at each time point were performed using Wilcoxon rank-sum tests, and within-donor changes (ΔT1–T0) were computed using Wilcoxon signed-rank tests.

Beta-diversity was assessed at the species level using Bray–Curtis dissimilarities computed via the distance() function from phyloseq (method = "bray"). Principal Coordinates Analysis (PCoA) was performed using the pcoa() function from the ape package (v5.8.1). Homogeneity of dispersion was evaluated using betadisper() from the vegan package (v2.6.10), with pairwise differences tested using Wilcoxon rank-sum tests. Differences in community composition were assessed by permutational multivariate analysis of variance (PERMANOVA) using the adonis2() function from vegan, stratifying by donor. Paired Bray–Curtis distances between T0 and T1 were also computed within donors to quantify individual-level compositional shifts, with statistical comparisons performed as described for α-diversity.

**Gut microbiota differential abundance analysis**

Community composition at baseline (T0) and follow-up (T1) were analysed to determine treatment-induced community shifts. For this purpose, gut microbiota differential abundance analysis was performed across four sample categories:

- A: Active group at baseline (T0)
- B: Placebo group at baseline (T0)
- C: Active group at follow-up (T1)
- D: Placebo group at follow-up (T1)

To visualise treatment effects on community composition, volcano plots were generated (**Supplementary Figure S1**). This type of scatter plot provides information on the magnitude of an effect (fold change on the x-axis) and the statistical significance of the effect (p-value on the y-axis). The cut-off for biological significance was set at a fourfold higher or lower abundance or enrichment in the active group as compared to the placebo group (|log2(fold change)| ≥ 2). Based on these results, it was opted to use Wilcoxon rank sum tests and Wilcoxon signed-rank tests, where appropriate, for statistical analysis. The cut-off for statistical significance was set at p ≤ 0.05. The obtained volcano plot classifies bacterial taxa into four different categories based on their abundance in compared conditions (**Supplementary Figure S1**): a) not statistically significant and not biologically relevant, b) biologically relevant, but not statistically significant, c) statistically significant, but not biologically relevant, and d) biologically and statistically significant.

**Supplementary Figure S1. Example of output generated with volcano plots**. Statistical significance was plotted in function of fold change, classifying bacterial taxa into four categories: not significant and not biologically relevant (grey), biologically relevant but not statistically significant (green), statistically significant, but not biologically relevant (blue), and biologically and statistically significant (red).


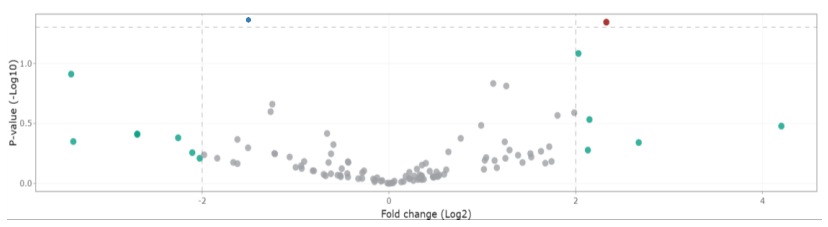


Volcano plots were generated to investigate the following:

- *Changes over time in community composition in both placebo (D versus B) and active (C versus A) groups*. Per study participant, fold changes were calculated from the bacterial abundances at both timepoints (T1/T0). For each bacterial taxon, the average fold change for a given taxon across the participants in a given group was calculated and presented in the volcano plot. To assess whether changes in the abundance of bacterial taxa on T1 and T0 were significant, Wilcoxon signed-rank test was used by comparing abundances before and after intervention.
- *Differences between active and placebo groups at the end-of-treatment period (C versus D).* For each bacterial taxon, averages of abundances were calculated across study participants in the placebo and active groups, from which then fold changes were generated (active/placebo ratio) and presented in the volcano plot. To assess whether differences in bacterial abundances between the active and the placebo groups were statistically significant, Wilcoxon rank sum test was used.

However, the latter comparison to assess treatment effects does not take into account either the temporal changes or the placebo effect expected to occur during the study. This is amplified by the observation that the gut microbiota is not constant in composition over time. Therefore, we also employed an approach consisting of comparisons of microbial abundances over the treatment period between the active and the placebo groups. A bacterial enrichment/inhibition is considered significant only if it exceeds the temporal changes and the placebo effect. A schematic representation of this approach is given in **Supplementary Figure S2.** Per participant and for each bacterial taxon, fold changes (ratio of abundance at follow-up (T1) versus baseline (T0)) were calculated for the active and the placebo groups. Subsequently, bacterial taxa for which the fold change was different in the active group from the placebo group were identified. To express the difference in increase or decrease of the various bacterial taxa over time between the placebo and active groups in the volcano plots, the difference in the log2 of the average fold change (across individuals) between the active and placebo groups was calculated for each bacterial taxon (log2 of average active sample’s fold change - log2 of average placebo sample’s fold change) (**Supplementary Figure S3**). P-values were calculated for each bacterial taxon, based on log2 of fold changes over time (T1/T0), using the Wilcoxon rank sum test. Because of the higher relevance of the latter method, where both temporal shifts and placebo effects are accounted for, we have only presented the results of this latter analysis in the main manuscript.

**Supplementary Figure S2. Schematic representation of the approach followed to assess active treatment effects.** Per participant and for each bacterial taxa (S1-Sx), fold changes (FC) were calculated (ratio of abundance at follow-up (T1) versus baseline (T0) (FC(T1/T0)) for the active and placebo groups. Then, bacterial taxa were identified for which the fold change was different in the active group from the placebo group.


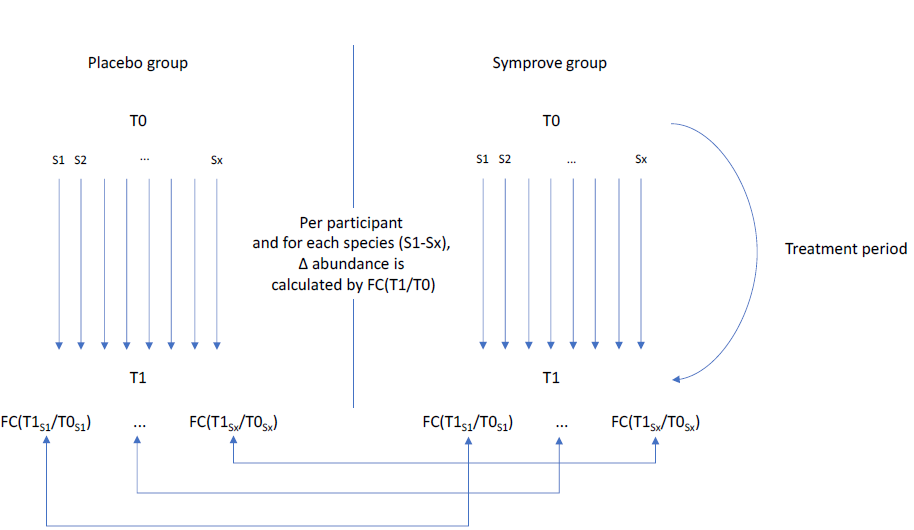


**Supplementary Figure S3. Equation 1: Calculation of differences in fold change between active and placebo groups for a given bacterial taxon.** Abbreviations: T0 = baseline, T1 = follow-up.


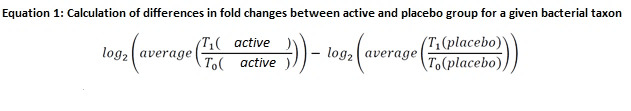


**SUPPLEMENTARY RESULTS**

**Supplementary Table S1. Baseline nutrition and physical exercise-related data.**

|  | **Active (n=35)** | **Placebo (n=33)** | **p** |
| --- | --- | --- | --- |
| **Physical activity**^^^  30 min aerobic exercise over the last 2 weeks  - none (%)  - once (%)  - twice (%)  - 3 to 5 times (%)  - 6 or more times (%)  - unspecified | 3 (8.6%)  1 (2.9%)  8 (22.9%)  11 (31.4%)  12 (34.3%)  0 (0.0%) | 4 (12.1%)  1 (3.0%)  6 (18.2%)  9 (27.3%)  12 (36.4%)  9 (3.0%) | 0.961 |
| **Food frequency^^^** |  |  |  |
| Meat - 1 large or two small pieces (125 gram)  - None (%)  - ≤ 1 per month (%)  - 1-3 per month (%)  - 1 per week (%)  - every other day (%)  - every day (%)  - unspecified (%) | 0 (0.0%)  3 (8.6%)  5 (14.3%)  5 (14.3%)  16 (45.7%)  5 (14.3%)  1 (2.9%) | 0 (0.0%)  2 (6.1%)  2 (6.1%)  9 (27.3%)  16 (48.5%)  3 (9.1%)  1 (3.0%) | 0.582 |
| Fish – 1 large or two small pieces (125 gram)  - None (%)  - ≤ 1 per month (%)  - 1-3 per month (%)  - 1 per week (%)  - every other day (%)  - every day (%)  - unspecified (%) | 0 (0.0%)  2 (5.7%)  1 (2.9%)  25 (71.4%)  6 (17.1%)  0 (0.0%)  1 (2.9%) | 0 (0.0%)  1 (3.0%)  3 (9.1%)  18 (54.5%)  10 (30.3%  0 (0.0%)  1 (3.0%) | 0.372 |
| Fresh Red Vegetables - 1/2 cup  - None (%)  - ≤ 1 per month (%)  - 1-3 per month (%)  - 1 per week (%)  - every other day (%)  - every day (%)  - unspecified (%) | 0 (0.0%)  1 (2.9%)  1 (2.9%)  9 (25.7%)  17 (48.6%)  7 (20.0%)  0 (0.0%) | 1 (3.0%)  0 (0.0%)  0 (0.0%)  9 (27.3%)  16 (48.5%)  6 (18.2%)  1 (3.0%) | 1.000 |
| Fresh non-Red Vegetables - 1/2 cup - None (%)  - ≤ 1 per month (%)  - 1-3 per month (%)  - 1 per week (%)  - every other day (%)  - every day (%)  - unspecified (%) | 0 (0.0%)  1 (2.9%)  1 (2.9%)  3 (8.6%)  12 (34.3%)  18 (51.4%)  0 (0.0%) | 0 (0.0%)  0 (0.0%)  0 (0.0%)  5 (15.2%)  12 (36.4%)  15 (45.5%)  1 (3.0%) | 0.746 |
| Fresh Fruit - 1 medium  - None (%)  - ≤ 1 per month (%)  - 1-3 per month (%)  - 1 per week (%)  - every other day (%)  - every day (%)  - unspecified (%) | 0 (0.0%)  1 (2.9%)  1 (2.9%)  6 (17.1%)  10 (28.6%)  17 (48.6%)  0 (0.0%) | 0 (0.0%)  0 (0.0%)  0 (0.0%)  4 (12.1%)  7 (21.2%)  21 (63.6%)  1 (3.0%) | 0.607 |
| Potatoes – 1 serving  - None (%)  - ≤ 1 per month (%)  - 1-3 per month (%)  - 1 per week (%)  - every other day (%)  - every day (%)  - unspecified (%) | 0 (0.0%)  1 (2.9%)  2 (5.7%)  7 (20.0%)  22 (62.9%)  3 (8.6%)  0 (0.0%) | 0 (0.0%)  2 (6.1%)  1 (3.0%)  12 (36.4%)  13 (39.4%)  4 (12.1%)  1 (3.0%) | 0.354 |
| Legumes - 1/2 cup  - None (%)  - ≤ 1 per month (%)  - 1-3 per month (%)  - 1 per week (%)  - every other day (%)  - every day (%)  - unspecified (%) | 0 (0.0%)  3 (8.6%)  4 (11.4%)  14 (40.0%)  9 (25.7%)  3 (8.6%)  2 (5.7%) | 2 (6.1%)  2 (6.1%)  4 (12.1%)  16 (48.5%)  7 (21.2%)  1 (3.0%)  1 (3.0%) | 0.729 |
| Cheese - 1 slice, 15 gram  - None (%)  - ≤ 1 per month (%)  - 1-3 per month (%)  - 1 per week (%)  - every other day (%)  - every day (%)  - unspecified (%) | 1 (2.9%)  1 (2.9%)  1 (2.9%)  7 (20.0%)  12 (34.3%)  11 (31.4%)  2 (5.7%) | 0 (0.0%)  3 (9.1%)  3 (9.1%)  9 (27.3%)  8 (24.2%)  9 (27.3%)  1 (3.0%) | 0.540 |
| Bread - 1 slice or 1 roll  - None (%)  - ≤ 1 per month (%)  - 1-3 per month (%)  - 1 per week (%)  - every other day (%)  - every day (%)  - unspecified (%) | 0 (0.0%)  1 (2.9%)  0 (0.0%)  1 (2.9%)  6 (17.1%)  27 (77.1%)  0 (0.0%) | 1 (3.0%)  0 (0.0%)  1 (3.0%)  2 (6.1%)  6 (18.2%)  21 (63.6%)  2 (6.1%) | 0.640 |
| Pasta - 1 cup  - None (%)  - ≤ 1 per month (%)  - 1-3 per month (%)  - 1 per week (%)  - every other day (%)  - every day (%)  - unspecified (%) | 0 (0.0%)  3 (8.6%)  7 (20.0%)  21 (60.0%)  4 (11.4%)  0 (0.0%)  0 (0.0%) | 1 (3.0%)  3 (9.1%)  7 (21.2%)  18 (54.5%)  3 (9.1%)  0 (0.0%)  1 (3.0%) | 0.988 |
| Grains - 1 cup  - None (%)  - ≤ 1 per month (%)  - 1-3 per month (%)  - 1 per week (%)  - every other day (%)  - every day (%)  - unspecified (%) | 2 (5.7%)  1 (2.9%)  3 (8.6%)  15 (42.9%)  8 (22.9%)  6 (17.1%)  0 (0.0%) | 1 (3.0%)  1 (3.0%)  5 (15.2%)  7 (21.2%)  12 (36.4%)  6 (18.2%)  1 (3.0%) | 0.463 |
| Soy - 1 cup  - None (%)  - ≤ 1 per month (%)  - 1-3 per month (%)  - 1 per week (%)  - every other day (%)  - every day (%)  - unspecified (%) | 7 (20.0%)  18 (51.4%)  6 (17.1%)  2 (5.7%)  1 (2.9%)  0 (0.0%)  1 (2.9%) | 7 (21.2%)  17 (51.5%)  3 (9.1%)  4 (12.1%)  0 (0.0%)  0 (0.0%)  2 (6.1%) | 0.698 |
| Nuts - 1/3 cup  - None (%)  - ≤ 1 per month (%)  - 1-3 per month (%)  - 1 per week (%)  - every other day (%)  - every day (%)  - unspecified (%) | 0 (0.0%)  7 (20.0%)  9 (25.7%)  7 (20.0%)  7 (20.0%)  5 (14.3%)  0 (0.0%) | 2 (6.1%)  8 (24.2%)  9 (27.3%)  6 (18.2%)  7 (21.2%)  0 (0.0%)  1 (3.0%) | 0.232 |
| Seeds - 1/3 cups  - None (%)  - ≤ 1 per month (%)  - 1-3 per month (%)  - 1 per week (%)  - every other day (%)  - every day (%)  - unspecified (%) | 4 (11.4%)  12 (34.3%)  7 (20.0%)  6 (17.1%)  3 (8.6%)  2 (5.7%)  1 (2.9%) | 7 (21.2%)  12 (36.4%)  8 (24.2%)  2 (6.1%)  2 (6.1%)  1 (3.0%)  1 (3.0%) | 0.675 |
| Water – 1 cup  - None (%)  - ≤ 1 per month (%)  - 1-3 per month (%)  - 1 per week (%)  - every other day (%)  - every day (%)  - 1 or 2 times per day (%)  - 3 or more times per day (%)  - unspecified (%) | 0 (0.0%)  0 (0.0%)  0 (0.0%)  0 (0.0%)  1 (2.9%)  4 (11.4%)  11 (31.4%)  19 (54.3%)  0 (0.0%) | 0 (0.0%)  0 (0.0%)  0 (0.0%)  0 (0.0%)  2 (6.1%)  4 (12.1%)  7 (21.2%)  19 (57.6%)  1 (3.0%) | 0.769 |
| Milk - 1 cup  - None (%)  - ≤ 1 per month (%)  - 1-3 per month (%)  - 1 per week (%)  - every other day (%)  - every day (%)  - 1 or 2 times per day (%)  - 3 or more times per day (%)  - unspecified (%) | 2 (5.7%)  2 (5.7%)  3 (8.6%)  3 (8.6%)  4 (11.4%)  13 (37.1%)  3 (8.6%)  5 (14.3%)  0 (0.0%) | 3 (9.1%)  7 (21.2%)  0 (0.0%)  4 (12.1%)  8 (24.2%)  5 (15.2%)  4 (12.1%)  1 (3.0%)  1 (3.0%) | 0.055 |
| Coffee - 1 cup  - None (%)  - ≤ 1 per month (%)  - 1-3 per month (%)  - 1 per week (%)  - every other day (%)  - every day (%)  - 1 or 2 times per day (%)  - 3 or more times per day (%)  - unspecified (%) | 0 (0.0%)  1 (2.9%)  0 (0.0%)  1 (2.9%)  4 (11.4%)  9 (25.7%)  11 (31.4%)  8 (22.9%)  1 (2.9%) | 1 (3.0%)  1 (3.0%)  2 (6.1%)  1 (3.0%)  1 (3.0%)  10 (30.3%)  9 (27.3%)  7 (21.2%)  1 (3.0%) | 0.769 |
| Diet soft-fizzy drinks - 1 can  - None (%)  - ≤ 1 per month (%)  - 1-3 per month (%)  - 1 per week (%)  - every other day (%)  - every day (%)  - unspecified (%) | 7 (20.0%)  23 (65.7%)  2 (5.7%)  2 (5.7%)  1 (2.9%)  0 (0.0%)  0 (0.0%) | 2 (6.1%)  21 (63.6%)  3 (9.1%)  3 (9.1%)  1 (3.0%)  2 (6.1%)  1 (3.0%) | 0.412 |
| Non-diet, soft fizzy drinks - 1 can  - None (%)  - ≤ 1 per month (%)  - 1-3 per month (%)  - 1 per week (%)  - every other day (%)  - every day (%)  - unspecified (%) | 9 (25.7%)  23 (65.7%)  0 (0.0%)  3 (8.6%)  0 (0.0%)  0 (0.0%) | 4 (12.1%)  22 (66.7%)  1 (3.0%)  3 (9.1%)  2 (6.1%)  1 (3.0%) | 0.309 |
| Olive oils - 1 spoon  - None (%)  - ≤ 1 per month (%)  - 1-3 per month (%)  - 1 per week (%)  - every other day (%)  - every day (%)  - unspecified (%) | 0 (0.0%)  3 (8.6%)  3 (8.6%)  7 (20.0%)  13 (37.1%)  8 (22.9%)  1 (2.9%) | 0 (0.0%)  2 (6.1%)  3 (9.1%)  6 (18.2%)  11 (33.3%)  10 (30.3%)  1 (3.0%) | 0.970 |
| Butter and margarine - 1 teaspoon  - None (%)  - ≤ 1 per month (%)  - 1-3 per month (%)  - 1 per week (%)  - every other day (%)  - every day (%)  - unspecified (%) | 1 (2.9%)  0 (0.0%)  1 (2.9%)  3 (8.6%)  5 (14.3%)  25 (71.4%)  0 (0.0%) | 0 (0.0%)  1 (3.0%)  1 (3.0%)  4 (12.1%)  11 (33.3%)  15 (45.5%)  1 (3.0%) | 0.151 |

Data presented as a number (percentage). Differences between groups were tested using Pearson Chi-square test or Fisher's exact test, where appropriate. Two-sided values of p<0.05 were considered statistically significant (in **bold**).

^ Based on locally validated nutrition and physical activity proforma.

**Supplementary Table S2. Evaluation of changes in Parkinson’s medication, nutrition, and physical activity-related data.**

|  | **Active (n=35)** | | | **Placebo (n=33)** | | |
| --- | --- | --- | --- | --- | --- | --- |
|  | **Pre-intervention** | **Post-intervention** | **p** | **Pre-intervention** | **Post-intervention** | **p** |
| **LEDD**  *Mean ± SD*  *Median (*25th and 75th percentiles)  *Minimum - Maximum* | 706.64 ± 477.89  550.00 (405.00– 1,018.00)  0.00 – 2,409.00 | 708.29 ± 483.16  550.00 (450.00-1,018.00)  0.00 – 2,409.00 | 0.684^a^ | 717.56 ± 449.53  575.00 (400.00 – 956.50)  180.00 – 2,412.05 | 729.08 ± 447.25  600.00 (450.00-956.50)  180.00 – 2,412.05 | 0.066^a^ |
| **Physical activity**  Frequency of 30 min aerobic exercise over the last 2 weeks^^^  - none (%)  - once (%)  - twice (%)  - 3 to 5 times (%)  - 6 or more (%)  - unspecified | 3 (8.6%)  1 (2.9%)  8 (22.9%)  11 (31.4%)  12 (34.3%)  0 (0.0%) | 3 (8.6%)  1 (2.9%)  5 (14.3%)  8 (22.9%)  17 (48.6%)  1 (2.9%) | 0.360^b^ | 4 (12.1%)  1 (3.0%)  6 (18.2%)  9 (27.3%)  12 (36.4%)  9 (3.0%) | 3 (9.1%)  3 (9.1%)  5 (15.2%)  6 (18.2%)  12 (36.4%)  4 (12.1%) | 0.564^b^ |
| **Food frequency^^^** |  |  |  |  |  |  |
| Meat - 1 large or two small pieces (125 gram)  - None (%)  - ≤ 1 per month (%)  - 1-3 per month (%)  - 1 per week (%)  - every other day (%)  - every day (%)  - unspecified (%) | 0 (0.0%)  3 (8.6%)  5 (14.3%)  5 (14.3%)  16 (45.7%)  5 (14.3%)  1 (2.9%) | 0 (0.0%)  2 (5.7%)  2 (5.7%)  11 (31.4%)  15 (42.95)  3 (8.6%)  2 (5.7%) | 1.000^b^ | 0 (0.0%)  2 (6.1%)  2 (6.1%)  9 (27.3%)  16 (48.5%)  3 (9.1%)  1 (3.0%) | 0 (0.0%)  1 (3.0%)  2 (6.1%)  5 (15.2%)  17 (51.5%)  4 (12.1%)  4 (12.1%) | 0.183^b^ |
| Fish – 1 large or two small pieces (125 gram)  - None (%)  - ≤ 1 per month (%)  - 1-3 per month (%)  - 1 per week (%)  - every other day (%)  - every day (%)  - unspecified (%) | 0 (0.0%)  2 (5.7%)  1 (2.9%)  25 (71.4%)  6 (17.1%)  0 (0.0%)  1 (2.9%) | 0 (0.0%)  0 (0.0%)  2 (5.7%)  23 (65.7%)  8 (22.9%)  0 (0.0%)  2 (5.7%) | 0.307^b^ | 0 (0.0%)  1 (3.0%)  3 (9.1%)  18 (54.5%)  10 (30.3%  0 (0.0%)  1 (3.0%) | 0 (0.0%)  1 (3.0%)  6 (18.2%)  14 (42.4%)  8 (24.2%)  0 (0.0%)  4 (12.1%) | 0.406^b^ |
| Fresh Red Vegetables - 1/2 cup  - None (%)  - ≤ 1 per month (%)  - 1-3 per month (%)  - 1 per week (%)  - every other day (%)  - every day (%)  - unspecified (%) | 0 (0.0%)  1 (2.9%)  1 (2.9%)  9 (25.7%)  17 (48.6%)  7 (20.0%)  0 (0.0%) | 0 (0.0%)  1 (2.9%)  2 (5.7%)  7 (20.0%)  12 (34.3%)  12 (34.3%)  1 (2.9%) | 0.471^b^ | 1 (3.0%)  0 (0.0%)  0 (0.0%)  9 (27.3%)  16 (48.5%)  6 (18.2%)  1 (3.0%) | 0 (0.0%)  0 (0.0%)  0 (0.0%)  8 (24.2%)  17 (51.5%)  4 (12.1%)  4 (12.1%) | 0.836^b^ |
| Fresh non-Red Vegetables - 1/2 cup–  - None (%)  - ≤ 1 per month (%)  - 1-3 per month (%)  - 1 per week (%)  - every other day (%)  - every day (%)  - unspecified (%) | 0 (0.0%)  1 (2.9%)  1 (2.9%)  3 (8.6%)  12 (34.3%)  18 (51.4%)  0 (0.0%) | 0 (0.0%)  0 (0.0%)  1 (2.9%)  7 (20.0%)  7 (20.0%)  19 (54.3%)  1 (2.9%) | 1.000^b^ | 0 (0.0%)  0 (0.0%)  0 (0.0%)  5 (15.2%)  12 (36.4%)  15 (45.5%)  1 (3.0%) | 0 (0.0%)  0 (0.0%)  2 (6.1%)  2 (6.1%)  16 (48.5%)  9 (27.3%)  4 (12.1%) | 0.148^b^ |
| Fresh Fruit - 1 medium  - None (%)  - ≤ 1 per month (%)  - 1-3 per month (%)  - 1 per week (%)  - every other day (%)  - every day (%)  - unspecified (%) | 0 (0.0%)  1 (2.9%)  1 (2.9%)  6 (17.1%)  10 (28.6%)  17 (48.6%)  0 (0.0%) | 0 (0.0%)  0 (0.0%)  2 (5.7%)  5 (14.3%)  7 (20.0%)  20 (57.1%)  1 (2.9%) | 0.241^b^ | 0 (0.0%)  0 (0.0%)  0 (0.0%)  4 (12.1%)  7 (21.2%)  21 (63.6%)  1 (3.0%) | 0 (0.0%)  0 (0.0%)  3 (9.1%)  2 (6.1%)  5 (15.2%)  19 (57.6%)  4 (12.1%) | 0.344^b^ |
| Potatoes – 1 serving  - None (%)  - ≤ 1 per month (%)  - 1-3 per month (%)  - 1 per week (%)  - every other day (%)  - every day (%)  - unspecified (%) | 0 (0.0%)  1 (2.9%)  2 (5.7%)  7 (20.0%)  22 (62.9%)  3 (8.6%)  0 (0.0%) | 0 (0.0%)  1 (2.9%)  2 (5.7%)  10 (28.6%)  16 (45.7%)  5 (14.3%)  1 (2.9%) | 0.613^b^ | 0 (0.0%)  2 (6.1%)  1 (3.0%)  12 (36.4%)  13 (39.4%)  4 (12.1%)  1 (3.0%) | 0 (0.0%)  2 (6.1%)  3 (9.1%)  8 (24.2%)  13 (39.4%)  3 (9.1%)  4 (12.1%) | 1.000^b^ |
| Legumes - 1/2 cup  - None (%)  - ≤ 1 per month (%)  - 1-3 per month (%)  - 1 per week (%)  - every other day (%)  - every day (%)  - unspecified (%) | 0 (0.0%)  3 (8.6%)  4 (11.4%)  14 (40.0%)  9 (25.7%)  3 (8.6%)  2 (5.7%) | 0 (0.0%)  2 (5.7%)  2 (5.7%)  18 (51.4%)  10 (28.6%)  0 (0.0%)  3 (8.6%) | 1.000^b^ | 2 (6.1%)  2 (6.1%)  4 (12.1%)  16 (48.5%)  7 (21.2%)  1 (3.0%)  1 (3.0%) | 1 (3.0%)  1 (3.0%)  8 (24.2%)  10 (30.3%)  8 (24.2%)  1 (3.0%)  4 (12.1%) | 0.868^b^ |
| Cheese - 1 slice, 15 gram  - None (%)  - ≤ 1 per month (%)  - 1-3 per month (%)  - 1 per week (%)  - every other day (%)  - every day (%)  - unspecified (%) | 1 (2.9%)  1 (2.9%)  1 (2.9%)  7 (20.0%)  12 (34.3%)  11 (31.4%)  2 (5.7%) | 0 (0.0%)  0 (0.0%)  2 (5.7%)  7 (20.0%)  13 (37.1%)  10 (28.6%)  3 (8.6%) | 0.341^b^ | 0 (0.0%)  3 (9.1%)  3 (9.1%)  9 (27.3%)  8 (24.2%)  9 (27.3%)  1 (3.0%) | 0 (0.0%)  2 (6.1%)  1 (3.0%)  6 (18.2%)  13 (39.4%)  7 (21.2%)  4 (12.1%) | 0.244^b^ |
| Bread - 1 slice or 1 roll  - None (%)  - ≤ 1 per month (%)  - 1-3 per month (%)  - 1 per week (%)  - every other day (%)  - every day (%)  - unspecified (%) | 0 (0.0%)  1 (2.9%)  0 (0.0%)  1 (2.9%)  6 (17.1%)  27 (77.1%)  0 (0.0%) | 0 (0.0%)  1 (2.9%)  0 (0.0%)  2 (5.7%)  5 (14.3%)  25 (71.4%)  2 (5.7%) | 1.000^b^ | 1 (3.0%)  0 (0.0%)  1 (3.0%)  2 (6.1%)  6 (18.2%)  21 (63.6%)  2 (6.1%) | 0 (0.0%)  0 (0.0%)  1 (3.0%)  2 (6.1%)  8 (24.2%)  17 (51.5%)  5 (15.2%) | 1.000^b^ |
| Pasta - 1 cup  - None (%)  - ≤ 1 per month (%)  - 1-3 per month (%)  - 1 per week (%)  - every other day (%)  - every day (%)  - unspecified (%) | 0 (0.0%)  3 (8.6%)  7 (20.0%)  21 (60.0%)  4 (11.4%)  0 (0.0%)  0 (0.0%) | 0 (0.0%)  2 (5.7%)  5 (14.3%)  24 (68.6%)  3 (8.6%)  0 (0.0%)  1 (2.9%) | 0.508^b^ | 1 (3.0%)  3 (9.1%)  7 (21.2%)  18 (54.5%)  3 (9.1%)  0 (0.0%)  1 (3.0%) | 0 (0.0%)  2 (6.1%)  10 (30.3%)  10 (30.3%)  6 (18.2%)  1 (3.0%)  4 (12.1%) | 0.261^b^ |
| Grains - 1 cup  - None (%)  - ≤ 1 per month (%)  - 1-3 per month (%)  - 1 per week (%)  - every other day (%)  - every day (%)  - unspecified (%) | 2 (5.7%)  1 (2.9%)  3 (8.6%)  15 (42.9%)  8 (22.9%)  6 (17.1%)  0 (0.0%) | 0 (0.0%)  2 (5.7%)  2 (5.7%)  13 (37.1%)  12 (34.3%)  5 (14.3%)  1 (2.9%) | 0.305^b^ | 1 (3.0%)  1 (3.0%)  5 (15.2%)  7 (21.2%)  12 (36.4%)  6 (18.2%)  1 (3.0%) | 0 (0.0%)  3 (9.1%)  6 (18.2%)  9 (27.3%)  10 (30.3%)  1 (3.0%)  4 (12.1%) | 0.207^b^ |
| Soy - 1 cup  - None (%)  - ≤ 1 per month (%)  - 1-3 per month (%)  - 1 per week (%)  - every other day (%)  - every day (%)  - unspecified (%) | 7 (20.0%)  18 (51.4%)  6 (17.1%)  2 (5.7%)  1 (2.9%)  0 (0.0%)  1 (2.9%) | 5 (14.3%)  16 (45.7%)  7 (20.0%)  5 (14.3%)  0 (0.0%)  0 (0.0%)  2 (5.7%) | 0.461^b^ | 7 (21.2%)  17 (51.5%)  3 (9.1%)  4 (12.1%)  0 (0.0%)  0 (0.0%)  2 (6.1%) | 2 (6.1%)  16 (48.5%)  5 (15.2%)  4 (12.1%)  0 (0.0%)  1 (3.0%)  5 (15.2%) | 0.145^b^ |
| Nuts - 1/3 cup  - None (%)  - ≤ 1 per month (%)  - 1-3 per month (%)  - 1 per week (%)  - every other day (%)  - every day (%)  - unspecified (%) | 0 (0.0%)  7 (20.0%)  9 (25.7%)  7 (20.0%)  7 (20.0%)  5 (14.3%)  0 (0.0%) | 1 (2.9%)  3 (8.6%)  9 (25.7%)  11 (31.4%)  5 (14.3%)  5 (14.3%)  1 (2.9%) | 0.877^b^ | 2 (6.1%)  8 (24.2%)  9 (27.3%)  6 (18.2%)  7 (21.2%)  0 (0.0%)  1 (3.0%) | 2 (6.1%)  6 (18.2%)  6 (18.2%)  8 (24.2%)  5 (15.2%)  2 (6.1%)  4 (12.1%) | 0.392^b^ |
| Seeds - 1/3 cups  - None (%)  - ≤ 1 per month (%)  - 1-3 per month (%)  - 1 per week (%)  - every other day (%)  - every day (%)  - unspecified (%) | 4 (11.4%)  12 (34.3%)  7 (20.0%)  6 (17.1%)  3 (8.6%)  2 (5.7%)  1 (2.9%) | 4 (11.4%)  12 (34.3%)  4 (11.4%)  9 (25.7%)  2 (5.7%)  1 (2.9%)  3 (8.6%) | 1.000^b^ | 7 (21.2%)  12 (36.4%)  8 (24.2%)  2 (6.1%)  2 (6.1%)  1 (3.0%)  1 (3.0%) | 3 (9.1%)  10 (30.3%)  9 (27.3%)  4 (12.1%)  2 (6.1%)  1 (3.0%)  4 (12.1%) | 0.358^b^ |
| Water – 1 cup  - None (%)  - ≤ 1 per month (%)  - 1-3 per month (%)  - 1 per week (%)  - every other day (%)  - every day (%)  - 1 or 2 times per day (%)  - 3 or more times per day (%)  - unspecified (%) | 0 (0.0%)  0 (0.0%)  0 (0.0%)  0 (0.0%)  1 (2.9%)  4 (11.4%)  11 (31.4%)  19 (54.3%)  0 (0.0%) | 0 (0.0%)  0 (0.0%)  0 (0.0%)  0 (0.0%)  1 (2.9%)  6 (17.1%)  12 (34.3%)  15 (42.9%)  1 (2.9%) | 0.307^b^ | 0 (0.0%)  0 (0.0%)  0 (0.0%)  0 (0.0%)  2 (6.1%)  4 (12.1%)  7 (21.2%)  19 (57.6%)  1 (3.0%) | 0 (0.0%)  0 (0.0%)  0 (0.0%)  1 (3.0%)  1 (3.0%)  4 (12.1%)  6 (18.2%)  17 (51.5%)  4 (12.1%) | 0.835^b^ |
| Milk - 1 cup  - None (%)  - ≤ 1 per month (%)  - 1-3 per month (%)  - 1 per week (%)  - every other day (%)  - every day (%)  - 1 or 2 times per day (%)  - 3 or more times per day (%)  - unspecified (%) | 2 (5.7%)  2 (5.7%)  3 (8.6%)  3 (8.6%)  4 (11.4%)  13 (37.1%)  3 (8.6%)  5 (14.3%)  0 (0.0%) | 1 (2.9%)  3 (8.6%)  1 (2.9%)  3 (8.6%)  3 (8.6%)  14 (40.0%)  8 (22.9%)  1 (2.9%)  1 (2.9%) | 0.916^b^ | 3 (9.1%)  7 (21.2%)  0 (0.0%)  4 (12.1%)  8 (24.2%)  5 (15.2%)  4 (12.1%)  1 (3.0%)  1 (3.0%) | 1 (3.0%)  6 (18.2%)  0 (0.0%)  2 (6.1%)  7 (21.2%)  9 (27.3%)  4 (12.1%)  0 (0.0%)  4 (12.1%) | 0.254^b^ |
| Coffee - 1 cup  - None (%)  - ≤ 1 per month (%)  - 1-3 per month (%)  - 1 per week (%)  - every other day (%)  - every day (%)  - 1 or 2 times per day (%)  - 3 or more times per day (%)  - unspecified (%) | 0 (0.0%)  1 (2.9%)  0 (0.0%)  1 (2.9%)  4 (11.4%)  9 (25.7%)  11 (31.4%)  8 (22.9%)  1 (2.9%) | 0 (0.0%)  1 (2.9%)  0 (0.0%)  1 (2.9%)  5 (14.3%)  9 (25.7%)  11 (31.4%)  6 (17.1%)  2 (5.7%) | 0.654^b^ | 1 (3.0%)  1 (3.0%)  2 (6.1%)  1 (3.0%)  1 (3.0%)  10 (30.3%)  9 (27.3%)  7 (21.2%)  1 (3.0%) | 1 (3.0%)  0 (0.0%)  1 (3.0%)  2 (6.1%)  3 (9.1%)  8 (24.2%)  9 (27.3%)  5 (15.2%)  4 (12.1%) | 1.000^b^ |
| Diet soft-fizzy drinks - 1 can  - None (%)  - ≤ 1 per month (%)  - 1-3 per month (%)  - 1 per week (%)  - every other day (%)  - every day (%)  - unspecified (%) | 7 (20.0%)  23 (65.7%)  2 (5.7%)  2 (5.7%)  1 (2.9%)  0 (0.0%)  0 (0.0%) | 7 (20.0%)  19 (54.3%)  4 (11.4%)  1 (2.9%)  3 (8.6%)  0 (0.0%)  1 (2.9%) | 0.217^b^ | 2 (6.1%)  21 (63.6%)  3 (9.1%)  3 (9.1%)  1 (3.0%)  2 (6.1%)  1 (3.0%) | 4 (12.1%)  16 (48.5%)  2 (6.1%)  3 (9.1%)  0 (0.0%)  4 (12.1%)  4 (12.1%) | 0.547^b^ |
| Non-diet, soft fizzy drinks - 1 can  - None (%)  - ≤ 1 per month (%)  - 1-3 per month (%)  - 1 per week (%)  - every other day (%)  - every day (%)  - unspecified (%) | 9 (25.7%)  23 (65.7%)  0 (0.0%)  3 (8.6%)  0 (0.0%)  0 (0.0%) | 7 (20.0%)  19 (54.3%)  6 (17.1%)  1 (2.9%)  1 (2.9%)  0 (0.0%)  1 (2.9%) | 0.361^b^ | 4 (12.1%)  22 (66.7%)  1 (3.0%)  3 (9.1%)  2 (6.1%)  1 (3.0%) | 6 (18.2%)  19 (57.6%)  1 (3.0%)  2 (6.1%)  0 (0.0%)  1 (3.0%)  4 (12.1%) | 0.148^b^ |
| Olive oils - 1 spoon  - None (%)  - ≤ 1 per month (%)  - 1-3 per month (%)  - 1 per week (%)  - every other day (%)  - every day (%)  - unspecified (%) | 0 (0.0%)  3 (8.6%)  3 (8.6%)  7 (20.0%)  13 (37.1%)  8 (22.9%)  1 (2.9%) | 1 (2.9%)  2 (5.7%)  2 (5.7%)  10 (28.6%)  13 (37.1%)  5 (14.3%)  2 (5.7%) | 0.363^b^ | 0 (0.0%)  2 (6.1%)  3 (9.1%)  6 (18.2%)  11 (33.3%)  10 (30.3%)  1 (3.0%) | 0 (0.0%)  1 (3.0%)  3 (9.1%)  6 (18.2%)  9 (27.3%)  10 (30.3%)  4 (12.1%) | 0.860^b^ |
| Butter and margarine - 1 spoon  - None (%)  - ≤ 1 per month (%)  - 1-3 per month (%)  - 1 per week (%)  - every other day (%)  - every day (%)  - unspecified (%) | 1 (2.9%)  0 (0.0%)  1 (2.9%)  3 (8.6%)  5 (14.3%)  25 (71.4%)  0 (0.0%) | 0 (0.0%)  0 (0.0%)  2 (5.7%)  4 (11.4%)  4 (11.4%)  24 (68.6%)  1 (2.9%) | 1.000^b^ | 0 (0.0%)  1 (3.0%)  1 (3.0%)  4 (12.1%)  11 (33.3%)  15 (45.5%)  1 (3.0%) | 0 (0.0%)  1 (3.0%)  1 (3.0%)  4 (12.1%)  10 (30.3%)  13 (39.4%)  4 (12.1%) | 1.000^b^ |

Data presented as mean ± standard deviation, median (25th and 75th percentiles), or number (percentage). Within-group changes were tested using:

1. Wilcoxon-Signed-rank test
2. Marginal homogeneity test

where appropriate. Two-sided values of p<0.05 were considered statistically significant (in **bold**).

^ Based on locally validated Nutrition and physical activity proforma

Abbreviations: LEDD: levodopa equivalent daily dose; SD: standard deviation.

**Supplementary Table S3. Adverse events and related withdrawals.**

| **Event** | **Active (n = 38)** | | **Placebo (n = 36)** | |
| --- | --- | --- | --- | --- |
|  | **n AE** | **n withdrawals** | **n AE** | **n withdrawals** |
| Abdominal bloating/discomfort | 3 | 0 | 2 | 1 |
| Flatulence | 2 | 0 | 1 | 0 |
| Diarrhoea | 0 | 0 | 2 | 0 |
| Gastritis | 1 | 1^a^ | 0 | 0 |
| Fall | 1 | 1 | 1 | 0 |
| Worsening of dyskinesia | 1 | 0 | 1 | 0 |
| Worsening of RLS | 0 | 0 | 1 | 0 |
| COVID-19 | 1 | 1 | 1 | 1 |
| Chest infection | 0 | 0 | 1 | 0 |
| Joint pain | 2 | 0 | 0 | 0 |
| Weight gain | 0 | 0 | 1 | 0 |
| **Total adverse events** | 11 | 3 | 11 | 2 |
| **Serious adverse events** | 0 | 0 | 0 | 0 |

^a^ probably related to the study intervention

Abbreviations: AE: adverse event. COVID-19: Coronavirus disease 2019; RLS: restless legs syndrome.

**Supplementary Figure S4.** **Gut microbiota α- and β-diversity at the species level show no differences between groups at either time point.** (A) Principal coordinates analysis (Bray–Curtis dissimilarity) of species-level relative microbiota profiles. PERMANOVA performed separately for each time point showed no significant differences between Active and Placebo groups at T0 (p = 0.400) or T1 (p = 0.579). (B) α-diversity metrics, including Observed richness (T0: p = 0.127; T1: p = 0.191), Shannon diversity (T0: p = 0.165; T1: p = 0.501), Inverse Simpson diversity (T0: p = 0.242; T1: p = 0.583), and Pielou’s evenness (T0: p = 0.694; T1: p = 0.969), did not differ significantly between groups at either time point. Abbreviations: ns = non-significant, T0= baseline; T1= follow-up.


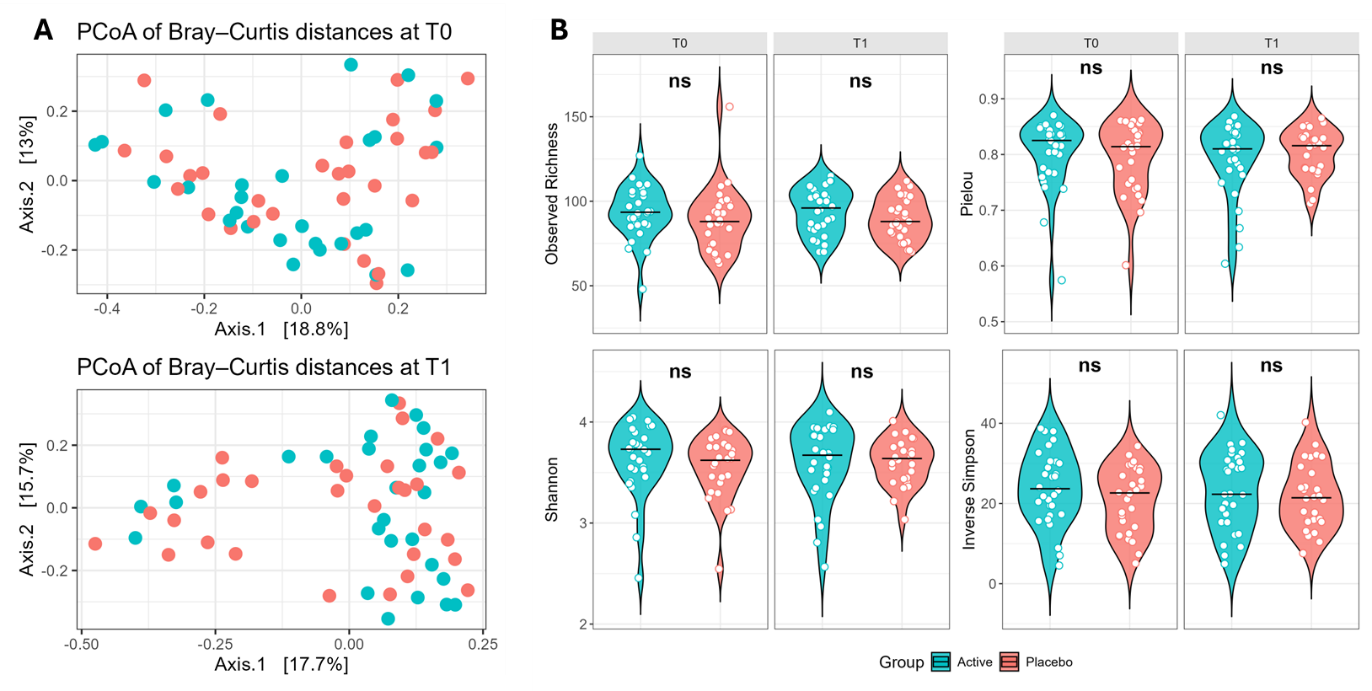


**Supplementary Figure S5. Gut microbiota α- and β-diversity at the species level show no microbial shifts.** (A) Principal coordinates analysis (Bray–Curtis dissimilarity) of species-level relative microbiota profiles with 95% confidence ellipses. PERMANOVA indicated no significant effects of Group (Active vs. Placebo; p = 1.000), Time (T0 vs. T1; p = 0.353), or their interaction (p = 0.514). Beta-dispersion analysis showed no differences in inter-individual variability across Group (p = 0.254), Time (p = 0.869), or Group × Time (p = 0.594), indicating that β-diversity patterns were not driven by changes in within-group heterogeneity. (B) Pairwise comparisons of distance to centroid showed no changes from T0 to T1 within groups (Placebo: p = 0.764; Active: p = 0.553) and no differences between groups at either time point (T0: p = 0.180; T1: p = 0.573), confirming stable within-group variability over time and between treatments. (C) Within-subject Bray–Curtis dissimilarity between T0 and T1 did not differ between groups (p = 0.562), although values were greater than zero within both Placebo (p < 0.001) and Active (p < 0.001) groups, suggesting natural temporal variability unrelated to treatment. (D) Δα-diversity (T1 – T0) for Observed richness, Shannon diversity, Inverse Simpson diversity, and Pielou’s evenness showed no significant differences between groups and no within-group deviations from baseline. Abbreviations: ns = non-significant, T0= baseline; T1= follow-up.


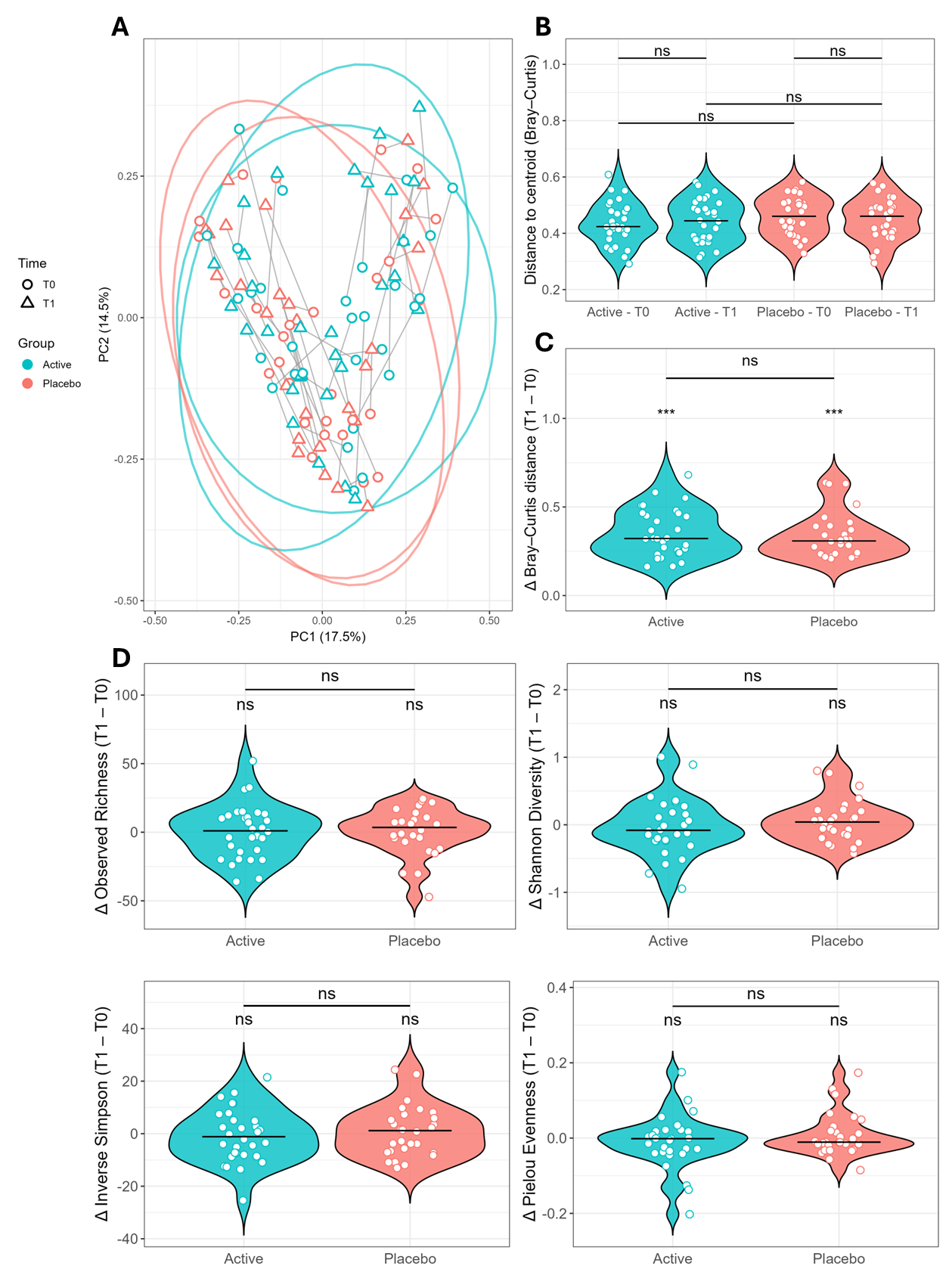


**Supplementary Figure S6. Between-group differences in normalised changes (****Δc/c_0_) of plasma levels of inflammatory cytokines.** Plasma levels of IFN-γ, TNF-α, IL-6, IL-8, and IL-10 were analysed using the Human ProInflammatory Panel 1 Kit from Meso Scale Discovery Mesoscale Discovery (MSD). Normalised delta = ((follow up value – baseline value)/ baseline value). Data presented as median and interquartile range. Data were analysed using the Mann-Whitney T test. *** p < 0.001. Abbreviations: IFNγ: interferon gamma; IL6: interleukin 6; IL8 interleukin 8; IL10: interleukin 10; TNFα: Tumor necrosis factor-α.


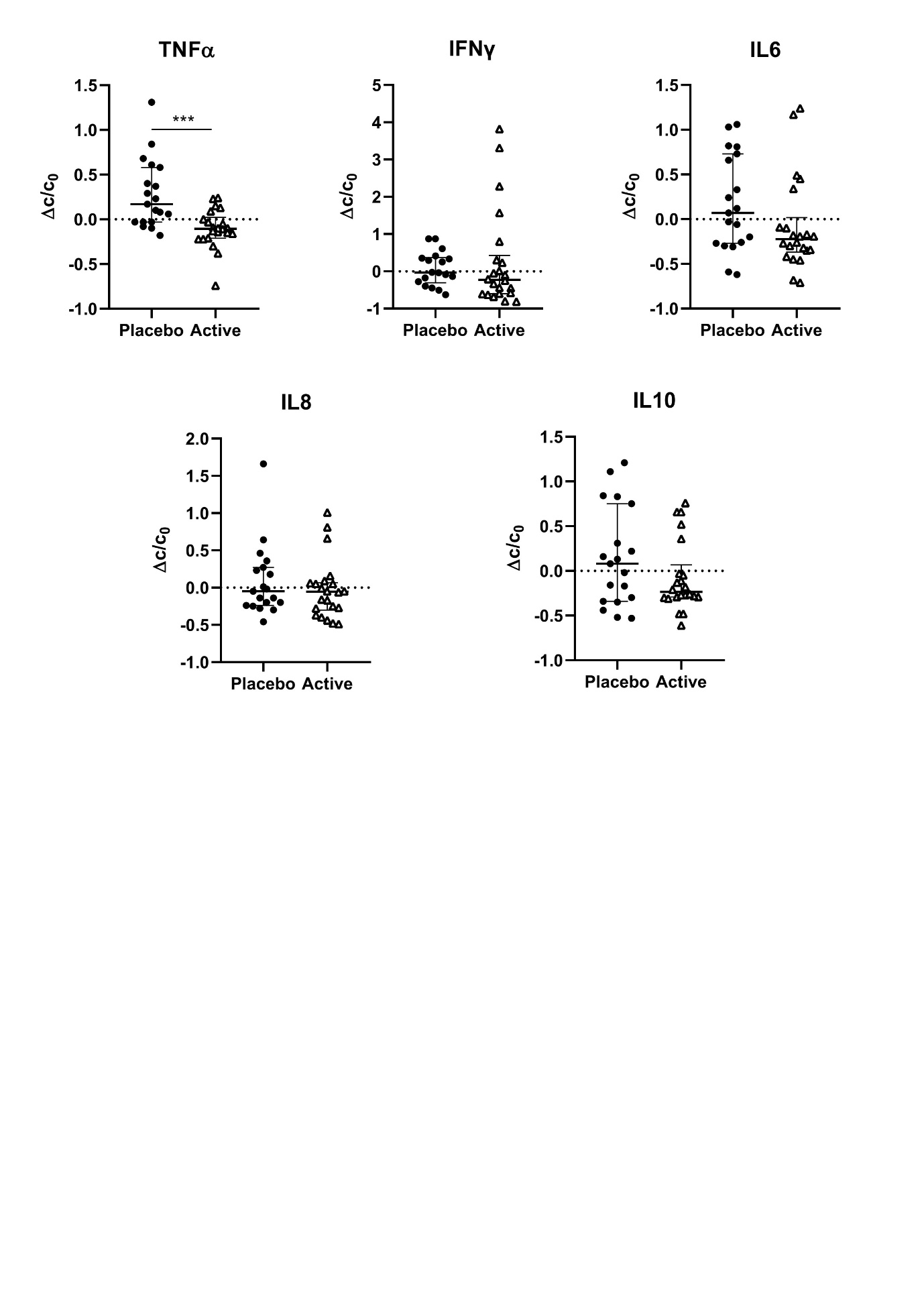


**References**

1. Boon N, Top EM, Verstraete W, Siciliano SD**.** Bioaugmentation as a tool to protect the structure and function of an activated-sludge microbial community against a 3-chloroaniline shock load. Appl Environ Microbiol. 2003;69(3):1511-20.

2. Hasan NA, Young BA, Minard-Smith AT, Saeed K, Li H, Heizer EM, et al. Microbial Community Profiling of Human Saliva Using Shotgun Metagenomic Sequencing. PLOS ONE. 2014;9(5):e97699.

3. Ottesen A, Ramachandran P, Reed E, White JR, Hasan N, Subramanian P, et al. Enrichment dynamics of Listeria monocytogenes and the associated microbiome from naturally contaminated ice cream linked to a listeriosis outbreak. BMC Microbiology. 2016;16(1):275.

4. Ponnusamy D, Kozlova EV, Sha J, Erova TE, Azar SR, Fitts EC, et al. Cross-talk among flesh-eating Aeromonas hydrophila strains in mixed infection leading to necrotizing fasciitis. Proc Natl Acad Sci U S A. 2016;113(3):722-7.

5. Lax S, Smith DP, Hampton-Marcell J, Owens SM, Handley KM, Scott NM, et al. Longitudinal analysis of microbial interaction between humans and the indoor environment. Science. 2014;345(6200):1048-52.

6. Borsini A, Di Benedetto MG, Giacobbe J, Pariante CM**.** Pro- and anti-inflammatory properties of interleukin (IL6) in vitro: relevance for major depression and for human hippocampal neurogenesis. Int J Neuropsychopharmacol. 2020.
